# CO_2_ Absorption by DBU-Based Protic Ionic Liquids: Basicity of Anion Dictates the Absorption Capacity and Mechanism

**DOI:** 10.3389/fchem.2018.00658

**Published:** 2019-01-17

**Authors:** Feixiang Gao, Zhen Wang, Pengju Ji, Jin-Pei Cheng

**Affiliations:** ^1^Department of Chemistry, Center of Basic Molecular Science, Tsinghua University, Beijing, China; ^2^School of Chemical and Environmental Engineering, Anyang Institute of Technology, Anyang, China; ^3^State Key Laboratory of Elemento-Organic Chemistry, Collaborative Innovation Center of Chemical Science and Engineering, Nankai University, Tianjin, China

**Keywords:** protic ionic liquids, basicity, CO_2_ absorption, linear correlation, absorption mechanism and capacity

## Abstract

PILs are promising solvent systems for CO_2_ absorption and transformations. Although previously tremendous work has been paid to synthesize functionalized PILs to achieve a high-performance absorption, the underlying mechanisms are far less investigated and still not clear. In this work, a series of DBU-based PILs, i.e., [DBUH][X], with anions of various basicities were synthesized. The basicities of the anions were accurately measured in [DBUH][OTf] or extrapolated from the known linear correlations. The apparent kinetics as well as the capacities for CO_2_ absorption in these PILs were studied systematically. The results show that the absorption rate and capacity in [DBUH][X] are in proportional to the basicity of PIL, i.e., a more basic PIL leads to a faster absorption rate and a higher absorption capacity. In addition, the spectroscopic evidences and correlation analysis indicate that the capacity and mechanism of CO_2_ absorption in [DBUH][X] are essentially dictated by the basicities of anions of these PILs.

## Introduction

Being considered as one of the major long-lived greenhouse gases that is responsible for the ever-increasing global warming phenomenon as well as ocean acidification (Jenkinson et al., 1999; Joos et al., 1999), carbon dioxide (CO_2_) has triggered tremendous research efforts in both academic and industry (Benson et al., 2009; Aresta et al., 2014; Goeppert et al., 2014; Sanna et al., 2014; Xia et al., 2018). To date a plethora of research attentions have been given to the processing, utilization and recycling of CO_2_, and one of the most fundamental research area among these studies is to design high-performance materials and develop a number of practical and efficient processes for CO_2_ capture and storage (CCS) (Haszeldine, 2009; Boot-Handford et al., 2014). Traditional chemical absorption of CO_2_ by aqueous solution of amines is a well-established process in this regard, and currently is an indispensable technology because of its low cost and good reactivity (Rao and Rubin, 2002). However, there have been growing concerns on the environmental issues associated with the use of aqueous amine solutions for CO_2_ absorption, such as high volatility and corrosive nature, etc. In addition, the degradation of amines during the absorption is also well-known, which significantly impairs the absorption capacity (Gouedard et al., 2012). Thus, discovering eco-friendly solvents/solvent systems or designing advanced materials as a potential replacement for the traditional CO_2_ absorption is highly desirable.

Ionic liquids (ILs) are composed of entirely ions and exhibit a number of properties that are significantly different from those of conventional molecular solvents. In addition, the cations and anions of ILs can be varied or functionalized, which may endow them with one or several favorable properties, such as negligible vapor pressure, low flammability, high conductivity, and good thermal stability (Welton, 1999; Hallett and Welton, 2011). As an important subset of ILs, protic ILs (PILs) can be conveniently prepared from stoichiometric neutralization between Brönsted acids and bases. Due to the presence of dissociable proton(s), compared with aprotic ILs (AILs), PILs exhibit a stronger hydrogen bond donicity and higher ionic conductivity under neat condition (Greaves and Drummond, 2008, 2015). Due to these merits that are distinctive from those of molecular solvents, ILs are labeled as green solvents (Rogers and Seddon, 2003) and have been extensively applied to catalysis, material and biological science as well as energy storage, etc. (van Rantwijk and Sheldon, 2007; Bideau et al., 2011; Watanabe et al., 2017).

Since Brennecke and co-worker demonstrated that CO_2_ has a good solubility in an imidazolium-based room temperature IL in 1999 (Blanchard et al., 1999), tremendous research efforts has been paid to utilize ILs or functionalized-ILs as media for physical and chemical CO_2_ absorptions during the past decades (Bates et al., 2002; Gurkan et al., 2010; Luo et al., 2014; Xia et al., 2018). These pioneer explorations on the CO_2_ absorptions in various ILs suggest that the absorption capacity and enthalpy are closely associated with the identity and structure of comprising cation and anion (Wang et al., 2010, 2012). By varying the structure of anions for these protic ILs, equimolar or even more than equimolar CO_2_ absorption has been achieved (Wang et al., 2011; Chen et al., 2016). Although a high absorption capacity of CO_2_ has been realized in ILs, the fundamental rules that govern the absorption mechanism and performances are still not clear, which may hamper a rational design and development of ILs in this respect. In addition, currently the rationales for the catalytic performance of PILs on CO_2_ absorption were almost entirely based on the acidity data determined in molecular solvents, such as water or DMSO (Wang et al., 2010; Yang et al., 2016). It is known from both experimental results and theoretical calculations that the acidity obtained in molecular solvents may not explain acid/base behavior in ILs satisfactorily (Mihichuk et al., 2011). For example, the absorption capacity of phorsphonium-based ILs, i.e., [R_4_P][X] (R = alkyl), was found sigmoidally (Yasuda and Watanabe, 2013) or linearly (Wang et al., 2012) correlated with the basicity of anion in molecular solvents. Therefore, it is sensible to use the acidity data determined in the ILs to assess CO_2_ absorption capacity, which may shed some lights on the intrinsic ability of ILs for CO_2_ absorption.

**Figure 1:**
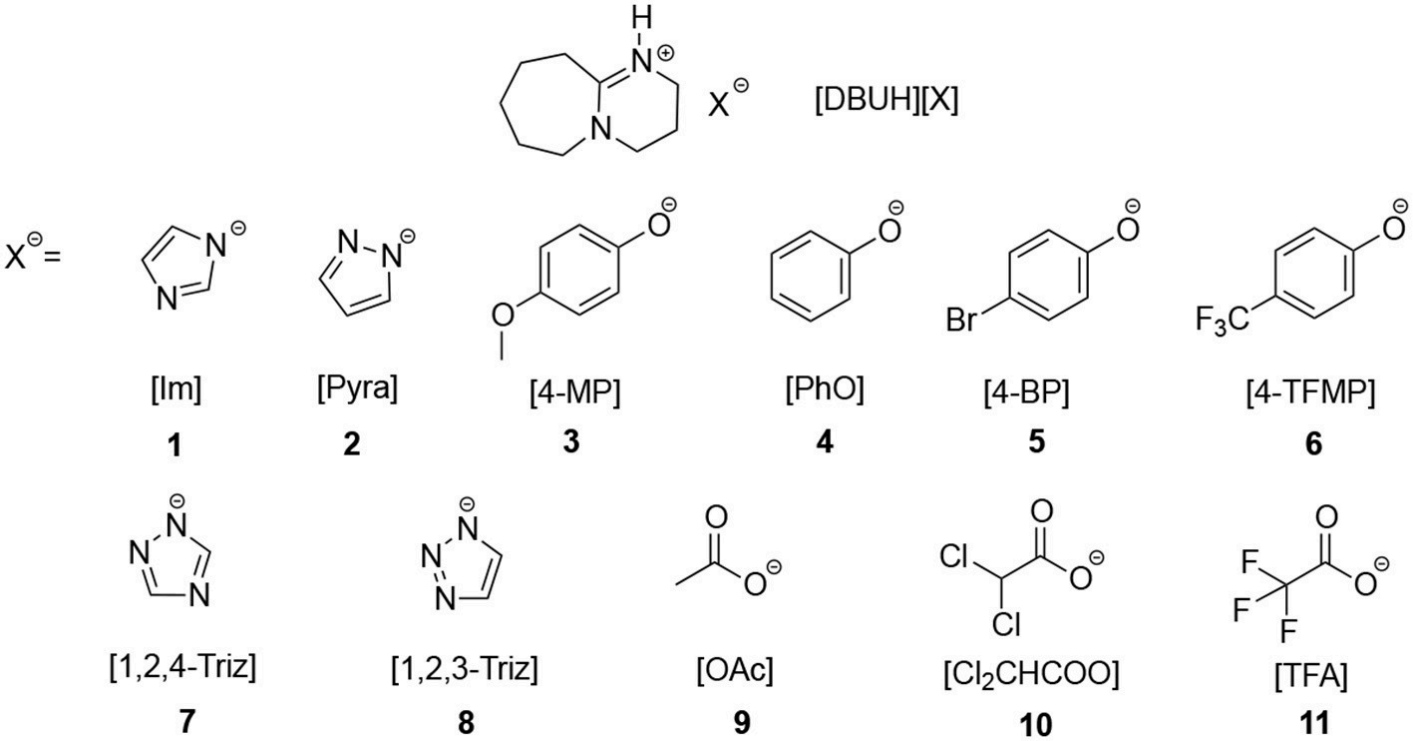
The structure of PILs [DBUH][X] involved in this work.

Superbase DBU-derived PILs [DBU = 1,5-diazabicyclo[5.4.0]-5-undecene] are promising solvent systems for the CO_2_ absorption, previous studies have shown that CO_2_ has a considerable solubility in these PILs (Losetty et al., 2017; Zhu et al., 2017). In this work, in order to systematically investigate the relationship between the absorption capacity and thermodynamic properties of ILs, firstly we synthesized 11 DBU-based PILs, i.e., [DBUH][X], whose anions (X^−^) are of different basicities (Figure 1), then the acidities of conjugated acids (HX) of these anions were determined or extrapolated from the known correlations obtained from previous study (Wang et al., 2018a). Next the apparent kinetics as well as capacity for CO_2_ absorption in [DBUH][X] were measured, whereby the relationship between the basicity of anion X^−^ and CO_2_ capture abilities of [DBUH][X] was established.

## Results and Discussion

### Basicity Scale for the Anion (X^−^) in [DBUH][X] (Figure 1)

**Figure 2:**
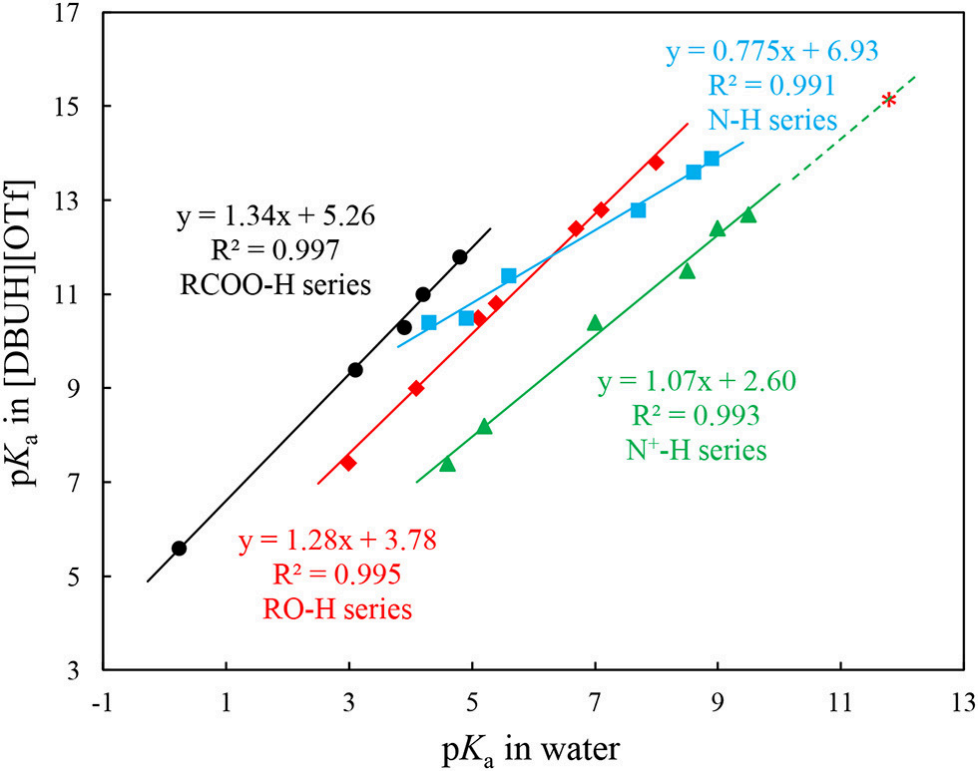
Correlations between p*K*_a_s of N-H (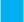), N-H^+^ (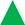), RO-H (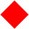), and carboxylic (RCOO-H, 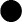) acids in [DBUH][OTf] and those in water. The red asterisk (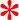) shows the extrapolated p*K*_a_ value of 15.1 for DBUH^+^ [${\text{p}K}_{\text{a}}^{\text{DBUH}+}$ in water = 11.7, (Kaupmees et al., 2014)] in [DBUH][OTf] from the linear correlation of N^+^-H series (Equation 1)^1^. Equations 1–4 were obtained from these linear correlations in Figure 2 and used to extrapolate the p*K*_a_s of anion precursors HX (**1**–**8**, Table 1) in [DBUH][OTf] (Table 1), in specific, the p*K*_a_s of **1**, **2**, **7**, and **8** are from Equation 2 and those of **3**–**6** are from Equation 3.

In our previous work, we have successfully measured the acidities for several series of commonly seen organic acids in a DBU-based PIL, i.e., [DBUH][OTf] (OTf ^−^ = triflate) (Wang et al., 2018a). The regression analyses show that the acidities of structural and electronic different organic substrates, i.e., RO-H, N-H, N^+^-H, and RCOO-H, linearly correlate with those in water. However, instead of forming a unified straight line, which is the case found for the analogous acidity correlation between a PIL EAN (ethylammonium nitrate) and water (Kanzaki et al., 2016), each individual series exhibits different slope and intercept (Figure 2) (Wang et al., 2018a). These linear acidity correlations between PILs and molecular solvent water are highly useful for accessing p*K*_a_ values of compounds in neat PILs that are difficult to determine due to the solvent leveling effect.

(1) $$\begin{array}{l} \text{For }{\text{N}}^{+}-H\text{ acids}:\text{p}K_{\text{a}}^{\left[\text{DBUH}\right]\left[\text{OTf}\right]}=1.07\text{p}K_{\text{a}}^{\text{water}}+2.60 \end{array}$$

(2) $$\begin{array}{l} \text{For N}-\text{H acids}:\text{p}K_{\text{a}}^{\left[\text{DBUH}\right]\left[\text{OTf}\right]}=0.775\text{p}K_{\text{a}}^{\text{water}}+6.93 \end{array}$$

(3) $$\begin{array}{l} \text{For O}-\text{H acids}:\text{p}K_{\text{a}}^{\left[\text{DBUH}\right]\left[\text{OTf}\right]}=1.28\text{p}K_{\text{a}}^{\text{water}}+3.78 \end{array}$$

(4) $$\begin{array}{l} \text{For COO}-\text{H acids}:\text{p}K_{\text{a}}^{\left[\text{DBUH}\right]\left[\text{OTf}\right]}=1.34\text{p}K_{\text{a}}^{\text{water}}+5.26 \end{array}$$

As shown in Figure 2, from the linear correlation of N^+^-H series, the acidity of protonated DBU, i.e., DBUH^+^ can be extrapolated as p*K*_a_ = 15.1 in neat [DBUH][OTf]^1^, which suggests that the acidities of these substrates with a p*K*_a_ close to and above this value (> ~14) are very difficult to be measured in neat [DBUH][OTf] by classical UV-vis spectroscopic approach (Yang et al., 2018). However, with the correlation equations provided in Figure 2, the basicities of a series of strong basic anions (**1**–**8**, Figure 1) for [DBUH][X] can be conveniently extrapolated from the linear correlation equations (Equations 1–4). Together with 3 experimentally determined basicities of less basic anions (**9**–**11**, Figure 1), a basicity scale that comprises totally 11 basicity values of anions (as expressed by the acidities of their anion precursors HX) involved in this work was established. Although these basicity values were acquired in [DBUH][OTf] and may be different from those in the PILs [DBUH][X], the relative basicity and nucleophilicity order of these anions are expected to be consistent between the DBU-based PILs. Table 1 lists the acidity of anion precursor (HX) in [DBUH][OTf], together with those available data in molecular solvents. As shown in Table 1, the basicity scale for the anions of [DBUH][X] covers 13 p*K* units and the basicities of these anions in [DBUH][OTf] are similar to those in DMSO but significantly greater than in water.

**Table 1:** The acidity of anion precursor (HX) in [DBUH][OTf] and the corresponding CO_2_ absorption capacity in [DBUH][X].

**Entry**	**[DBUH][X]**	***^a^*p*K*_**a**_^**[DBUH][OTf]**^ (HX)**	***^a^, ^b^*p*K*_**a**_^**water**^ (HX)**	***^a^, ^b^*p*K*_**a**_^**DMSO**^ (HX)**	**CO_**2**_ absorption*^c^***
1	[DBUH][Im]	18.2*^d^*	14.5	18.6	1.19
2	[DBUH][Pyra]	17.7*^d^*	13.9	19.8	1.15
3	[DBUH][4-MP]	16.8*^d^*	10.2	19.1	0.90
4	[DBUH][PhO]	16.6*^d^*	10.0	18.0	0.84
5	[DBUH][4-BP]	15.8*^d^*	9.4	16.4	0.70
6	[DBUH][4-TFMP]	14.9*^d^*	8.7	15.2	0.61
7	[DBUH][1,2,4-Triz]	14.7*^d^*	10.0	14.7_5_	0.55
8	[DBUH][1,2,3-Triz]	14.3*^d^*	9.5	13.9	0.52
9	[DBUH][OAc]	11.8*^e^*	4.7_5_	12.5	0.24
10	[DBUH][Cl2CHCOO]	7.6*^e^*	1.3_5_	6.4	0.06
11	[DBUH][TFA]	5.6*^e^*	0.23	3.6	0.05

a The conjugated acid HX of the corresponding anion in [DBUH][X].

b pK_a_ data is from: Internet Bond-energy Databank (iBonD), ibond.chem.tsinghua.edu.cn or ibond.nankai.edu.cn.

c Mol CO_2_ per mol PIL, the experiments were conducted at constant 25°C under atmospheric pressure, SD = ± 0.05, which is based on 3 individual absorption experiments.

d Extrapolated values obtained from the corresponding linear correlations (Figure 2 and Equations 1–4).

e *Determined experimentally, SD ≤ ± 0.05 pK units*.

### CO_2_ Absorption in [DBUH][X]

**Figure 3:**
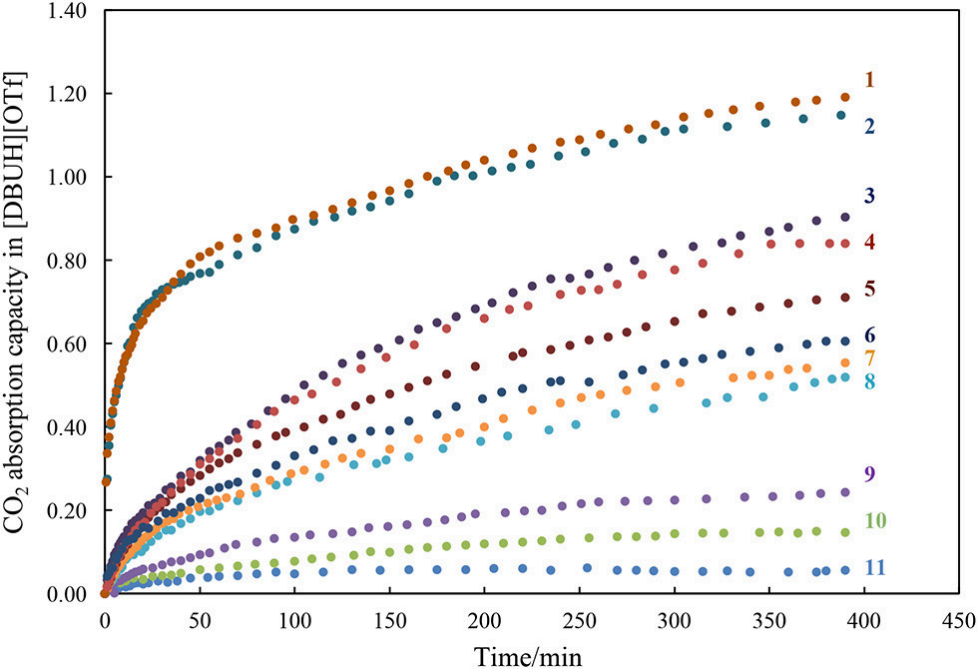
The kinetic profiles of CO_2_ absorption in the PIL [DBUH][X] (**1**–**11**, Table 1).

With the basicity scale for the anions in our hands, next we systematically measured the apparent kinetic of CO_2_ absorption in [DBUH][X], with a control of temperature at constant 25°C by a thermostat and the measurement was performed under atmosphere pressure. The CO_2_ absorption capacity in the individual [DBUH][X] is also determined as the molar ratio between the maximum amount of CO_2_ absorbed and of [DBUH][X] used (Table 1). It is worth noting that the viscosity of [DBUH][X] increased with the increasing amount of CO_2_ absorbed, forming a gel-like liquid which leads to a relatively large standard deviation (SD = ±0.05 of the absorption molar ratio). However, the volume of PILs did not have an obvious increase through CO_2_ uptake, which is in line with the previous reported (Firaha and Kirchner, 2014). Figure 3 shows the apparent kinetic profile of CO_2_ absorption in these PILs, in general, the rates for CO_2_ absorption in [DBUH][X] are slower than those observed in phosphonium-based aprotic ILs ([PR_4_][X]), probably due to the hydrogen bonding between DBUH^+^ and X^−^ in [DBUH][X], which makes anions less reactive toward CO_2_ than those in [PR_4_][X] (Wang et al., 2011). As also can be seen from Figure 3, the rate of CO_2_ absorption is faster in the [DBUH][X] with a more basic anion X^−^ than in those with less basic ones. In addition, as shown from Table 1, the maximum absorption capacity of [DBUH][X] decreases with the decreasing basicity of anions. For examples, the amount of CO_2_ uptake for the most basic [DBUH][Im] (**1**) is about twice as much as that for the less basic [DBUH][4-TFMP] (**6**). On the other hand, there is a sharp decrease in CO_2_ absorption capacity in these weakly basic PILs (**8**–**11**), as for the least basic PILs, such as [DBUH][TFA] (**11**) and [DBUH][Cl_2_CHCOO] (**10**), they both have a very limited absorption ability, despite of their obvious basicity difference.

**SCHEME 1:**
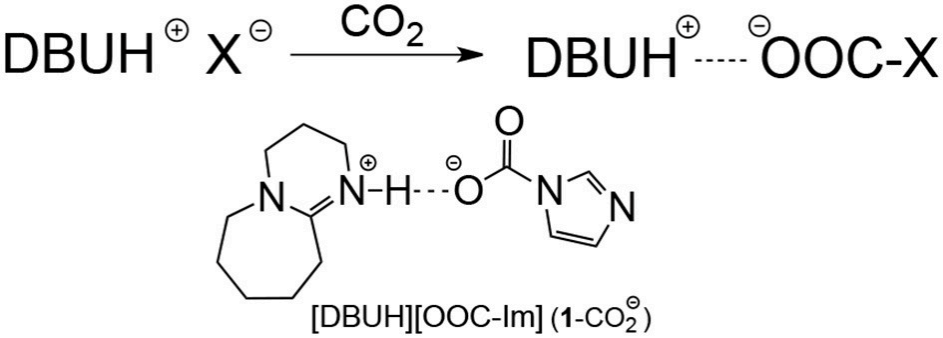
Chemical absorption mechanism in the strong basic [DBUH][X] and the possible formation of hydrogen bonded complex (**1**-${\text{CO}}_{2}^{-}$) after CO_2_ uptake in [DBUH][Im] (**1**).

In order to understand the absorption mechanism, the CO_2_ absorption in [DBUH][X] was monitored by NMR and IR spectroscopies. The ^13^C NMR and IR spectra for each [DBUH][X] before and after CO_2_ absorption were recorded and compared (Supplementary Material provides full characterizations, herein only a representative example is presented). Spectroscopic results show that there is no change in both ^13^C NMR and IR spectra before and after the absorption for [DBUH][X] with a relatively weak basic anion (**8**–**11**, Table 1, for details, see Supplementary Material). Presumably, this is due to the basicities of anions for these PILs are too weak to react with CO_2_ to form the corresponding carboxylates (Scheme 1, vide infra), therefore a physical absorption mechanism likely dominates in these PILs (Izgorodina et al., 2015).

**Figure 4:**
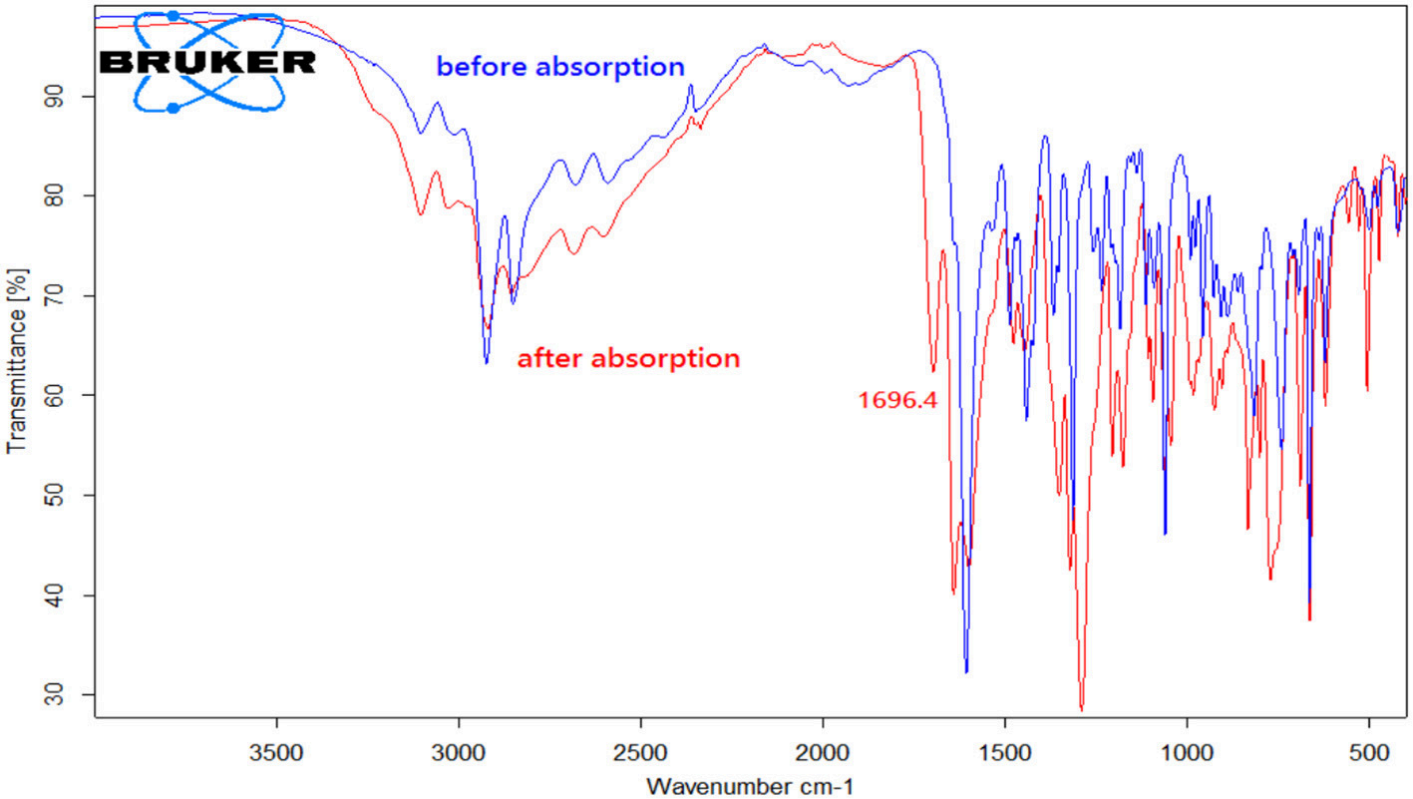
IR spectra of [DBUH][Im] (**1**) before and after CO_2_ absorption.

**Figure 5:**
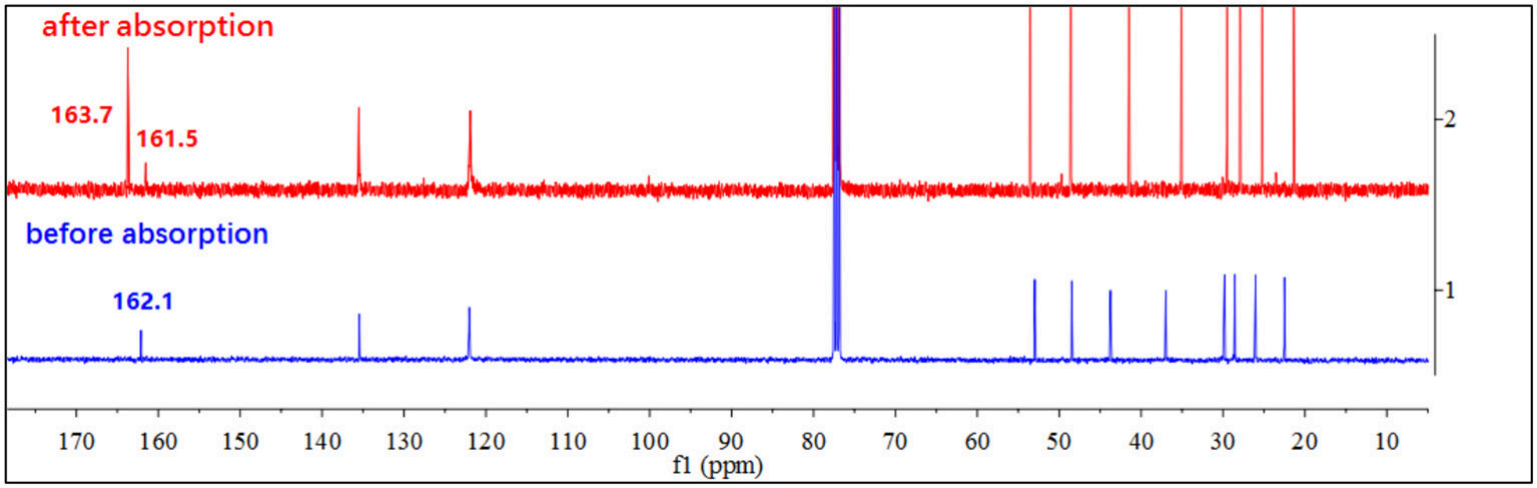
^13^C NMR spectra of [DBUH][Im] (**1**) before and after CO_2_ absorption.

By contrast, the [DBUH][X] with relatively strong basic anions (**1**–**7**, Figure 1) clearly exhibit a different absorption mechanism as revealed by the results from ^13^C NMR and IR spectra. Compared with those before CO_2_ uptake, the IR and ^13^C NMR spectra of **1**–**7** after CO_2_ uptake show a new peak at ~1,700 cm^−1^ (C = O stretching) and a new signal at ~163 ppm which are characteristic of carbonyl carbons in carbamates or carbonates, respectively (Figures 4, 5, Supplementary Material). As a representative example, the IR spectrum of [DBUH][Im] (**1**) after absorption shows a distinctive peak at 1,696 cm^−1^, in addition, a new signal at 161.5 ppm was observed in ^13^C NMR spectrum (Figures 4, 5).

These spectra results indicate that the mechanism for [DBUH][X] with a relatively strong basic anion (**1**–**7**) follows a chemical absorption mechanism (Wang et al., 2011; Chen et al., 2016). As illustrated in Scheme 1, the anions of **1**–**7** react with CO_2_ through a nucleophilic attack process which yields the corresponding carboxylate adducts [DBUH][OOC-X], and the rate of forming carboxylate is in proportion to the basicity of anion which, under most circumstances, is paralleled with its nucleophilicity (Figure 3)^2^.

**Figure 6:**
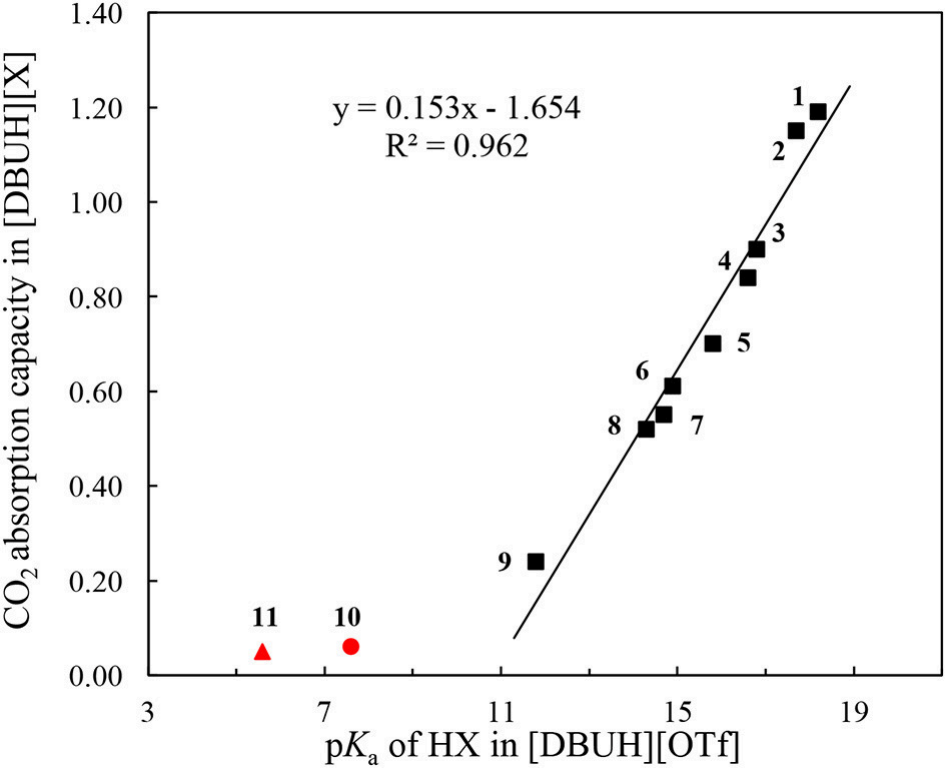
The correlation between CO_2_ absorption capacity in a series of [DBUH][X] and the acidity of the anion precursor HX (**1**–**9**) in [DBUH][OTf] (*R*^2^ = 0.962). The outliners are [DBUH][TFA] (**11**, 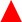) and [DBUH][Cl_2_CHCOO] (**10**, 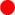).

Linear correlation can be a useful tool to reveal the underlying factors that govern the absorption kinetic and mechanism. In this connection, the correlation between the absorption capacity of [DBUH][X] and the basicity of anion in [DBUH][OTf] was performed. As shown in Figure 6, one can clearly notice a transition of CO_2_ absorption capacity which is regulated by the basicity of anion. Specifically, there is a fairly good linear relationship (*R*^2^ = 0.962) between the absorption capacity in [DBUH][X] (**1**–**9**) and the basicity of anion in [DBUH][OTf], excluding the data points of very weakly basic [DBUH][TFA] (**11**) and [DBUH][Cl_2_CHCOO] (**10**). Combined with the evidences from the spectroscopic studies, we can conclude with confidence that a chemical absorption mechanism occurs in the [DBUH][X] (**1**–**7**) whose anion precursor HX has a p*K*_a_ > 15 in [DBUH][OTf], while the CO_2_ absorption follows a physical absorption mechanism in [DBUH][X] (**10**–**11**) with an anion precursor's p*K*_a_ < 10. Presumably, a mixed chemical and physical mechanism occurs in these PILs with an anion precursor p*K*_a_ between 10 and 15, such as the CO_2_ absorption in **8** and **9**. The quantum chemical calculations would be an ideal tool for the mechanism elucidation of CO_2_ absorption in the PILs, however, currently some crucial physical and chemical parameters, such as dielectric constants, etc., for these PILs [DBUH][X] are not yet available, which hampers a detailed and reliable theoretical calculation for the CO_2_ absorption mechanism study in these PILs. It is worth noting that, by contrast, a similar correlation between absorption capacity and p*K*_a_ for precursor (**1**–**9**) of anion in molecular solvents, such as water and DMSO exhibits an inferior linear correlation (*R*^2^ = 0.874 and 0.898, respectively, Figures S36, S37), which implies that the bond energetic data obtained in molecular solvents, though relatively abundant and well-established, may not be suitable to interpret the experimental observations in ILs. Therefore, cares should be taken when one attempts to utilize the thermodynamic parameters measured in *molecular solvents* to disclose the governing factors for the gas absorptions in *PILs*.

## Experimental

### Chemicals and CO_2_ Gas

All the chemicals and solvents were purchased from commercially available sources, and used directly without further purification except otherwise noted. DBU was also from commercially available sources, but purified from multiple reduced pressure distillation. CO_2_ gas was provided by the Linde Industrial Gases with a purity of >99.9995%, and was directly generated into [DBUH][X] for the CO_2_ absorption.

### Instrumentations

The IR spectra were recorded on a Bruker Tensor II FT-IR instrument. The ^1^H NMR and ^13^C NMR spectra were recorded on a Bruker AVANCE III HD 400 MHz spectrometer. The water content was determined by a Mettler Toledo V20S compact volumetric Karl-Fischer titrator. UV-vis spectra were obtained from an Agilent Cary 100 machine with the control of temperature at constant 25°C.

### Preparation of [DBUH][X]

[DBUH][X] were synthesized by direct equal molar neutralization reactions between DBU and acids under neat condition or in methanol, Supplementary Material provides the detailed synthetic procedures. The structure of [DBUH][X] were confirmed by NMR and IR spectroscopies. The water content of prepared [DBUH][X] varies from 100 to 300 ppm, which was determined by a Karl-Fisher titration machine. It is worth noting that the water content of [DBUH][X] has only a limited effect on the CO_2_ absorption in the range of 100 to 300 ppm, as the comparison experiments showed that nearly the identical amount of CO_2_ was absorbed by the [DBUH][X] with a water content of 100 or 300 ppm.

[DBUH][Im] (**1**): ^1^H NMR (400 MHz, CDCl_3_) δ 11.49 (br, 1H), 7.64 (s, 1H), 7.06 (s, 2H), 3.57–2.96 (m, 6H), 2.39 (s, 2H), 1.90–1.74 (m, 2H), 1.72–1.32 (m, 6H); ^13^C NMR (101 MHz, CDCl_3_) δ 162.1, 135.5, 122.0, 53.0, 48.5, 43.7, 37.0, 29.8, 28.6, 26.0, 22.5 ppm; IR (neat): 3147, 2923, 2850, 2779, 2742, 2671, 2349, 1999, 1608 cm^−1^;

[DBUH][Pyra] (**2**): ^1^H NMR (400 MHz, CDCl_3_) δ 12.63 (br, 0.49H), 7.53 (d, *J* = 1.8 Hz, 2H), 6.24 (t, *J* = 1.8 Hz, 1H), 3.26 (t, *J* = 5.5 Hz, 2H), 3.20–3.08 (m, 4H), 2.42–2.26 (m, 2H), 1.82–1.70 (m, 2H), 1.68–1.41 (m, 6H); ^13^C NMR (101 MHz, CDCl_3_) δ 162.1, 133.3, 104.3, 52.9, 48.4, 43.5, 36.8, 29.7, 28.4, 25.8, 22.3 ppm; IR (neat): 3142, 3049, 2924, 2850, 2675, 2350, 1900, 1607 cm^−1^;

[DBUH][4-MP] (**3**): ^1^H NMR (400 MHz, CDCl_3_) δ 12.35 (br, 0.74H), 6.76–6.64 (m, 4H), 3.69 (s, 3H), 3.29–3.16 (m, 6H), 2.50–2.40 (m, 2H), 1.84–1.74 (m, 2H), 1.66–1.49 (m, 6H); ^13^C NMR (101 MHz, CDCl_3_) δ 163.0, 154.1, 151.5, 116.8, 114.7, 55.9, 53.1, 48.4, 42.0, 35.1, 29.6, 28.1, 25.5, 21.8 ppm; IR (neat): 2926, 2852, 2666, 2510, 2349, 2109, 1606 cm^−1^;

[DBUH][PhO] (**4**): ^1^H NMR (400 MHz, CDCl_3_) δ 13.26 (br, 0.85H), 7.18–7.05 (m, 2H), 6.88–6.75 (m, 2H), 6.66 (t, *J* = 7.3 Hz, 1H), 3.31–3.25 (m, 2H), 3.23 (t, *J* = 6.2 Hz, 4H), 2.56–2.37 (m, 2H), 1.89–1.75 (m, 2H), 1.72–1.47 (m, 6H). ^13^C NMR (101 MHz, CDCl_3_) δ 163.2, 160.8, 129.3, 117.0, 116.7, 53.2, 48.4, 41.8, 35.0, 29.6, 28.1, 25.4, 21.7 ppm; IR (neat): 3047, 2925, 2852, 2684, 2455, 2349, 2094, 1816, 1581 cm^−1^;

[DBUH][4-BP] (**5**): ^1^H NMR (400 MHz, CDCl_3_) δ 7.09 (d, *J* = 8.7 Hz, 2H), 6.67–6.56 (m, 2H), 3.24 (dd, *J* = 12.4, 6.9 Hz, 6H), 2.61–2.48 (m, 2H), 1.88–1.74 (m, 2H), 1.68–1.45 (m, 6H); ^13^C NMR (101 MHz, CDCl_3_) δ 164.7, 161.3, 131.8, 118.9, 107.4, 53.7, 48.4, 39.6, 33.3, 29.2, 27.3, 24.6, 20.4 ppm; IR (neat): 2926, 2853, 2449, 2349, 2101, 1860, 1640 cm^−1^;

[DBUH][4-TFMP] (**6**): ^1^H NMR (400 MHz, CDCl_3_) δ 12.51 (br, 1H), 7.31 (d, *J* = 8.6 Hz, 2H), 6.72 (d, *J* = 8.6 Hz, 2H), 3.33–3.23 (m, 6H), 2.60–2.52 (m, 2H), 1.92–1.81 (m, 2H), 1.73–1.54 (m, 6H); ^13^C NMR (101 MHz, CDCl_3_) δ 166.8, 164.3, 126.8(q, *J*_C−F_ = 3.7 Hz), 125.6 (q, *J* = 270.0 Hz), 117.2, 116.8 (q, *J*_C−F_ = 32.1 Hz), 53.6, 48.5, 40.5, 33.9, 29.4, 27.7, 25.0, 21.0 ppm; IR (neat): 2930, 2859, 2675, 2349, 2100, 1856, 1640, cm^−1^;

[DBUH][1,2,4-Triz] (**7**): ^1^H NMR (400 MHz, CDCl_3_) δ 14.81 (br, 1H), 8.06 (s, 2H), 3.48–3.14 (m, 6H), 2.78–2.41 (m, 2H), 1.93–1.82 (m, 2H), 1.72–1.54 (m, 6H); ^13^C NMR (101 MHz, CDCl_3_) δ 164.2, 148.3, 53.6, 48.5, 40.6, 34.2, 29.4, 27.7, 25.0, 21.0 ppm; IR (neat): 3078, 2925, 2855, 2473, 2350, 2072, 1898, 1638, 1611 cm^−1^;

[DBUH][1,2,3-Triz] (**8**): ^1^H NMR (400 MHz, CDCl_3_) δ 12.60 (br, 1H), 7.59 (d, *J* = 19.6 Hz, 2H), 3.63–3.02 (m, 6H), 2.90–2.56 (m, 2H), 1.93–1.80 (m, 2H), 1.58 (dd, *J* = 25.3, 3.8 Hz, 6H); ^13^C NMR (101 MHz, CDCl_3_) δ 165.1, 130.0, 53.8, 48.4, 39.1, 33.0, 29.1, 27.2, 24.4, 20.1 ppm; IR: 3234, 3101, 2926, 2857, 2672, 2117, 1881, 1637 cm^−1^;

[DBUH][OAc] (**9**): ^1^H NMR (400 MHz, CDCl_3_) δ 3.46–3.26 (m, 6H), 2.81 (d, *J* = 5.4 Hz, 2H), 1.99–1.85 (m, 5H), 1.68 (m, 4H), 1.60 (m, 2H); ^13^C NMR (101 MHz, CDCl_3_) δ 177.4, 165.6, 53.8, 48.3, 37.7, 31.6, 28.8, 26.8, 24.4, 23.9, 19.5 ppm; IR (neat): 3249, 2925, 2859, 2670, 2349, 2103, 1887, 1641, cm^−1^;

[DBUH][Cl_2_CHCOO] (**10**): ^1^H NMR (400 MHz, CDCl_3_) δ 11.76 (br, 1H), 5.80 (s, 1H), 3.60–3.18 (m, 6H), 2.76 (d, *J* = 6.0 Hz, 2H), 2.08–1.78 (m, 2H), 1.75–1.55 (m, 6H); ^13^C NMR (101 MHz, CDCl_3_) δ 168.3, 166.2, 70.0, 54.4, 48.7, 38.3, 32.3, 29.1, 26.9, 24.2, 19.7 ppm; IR (neat): 3229, 2929, 2859, 2802,1632, 1377 cm^−1^;

[DBUH][TFA] (**11**): ^1^H NMR (400 MHz, CDCl_3_) δ 11.23 (br, 1H), 3.48–3.37 (m, 4H), 3.32 (dd, *J* = 7.8, 5.9 Hz, 2H), 2.79–2.65 (m, 2H), 2.00–1.89 (m, 2H), 1.63 (ddd, *J* = 14.7, 8.4, *J* = 5.4 Hz, 6H); ^13^C NMR (101 MHz, CDCl_3_) δ: 166.2, 161.3, 117.1, 54.3, 48.5, 38.1, 32.2, 28.9, 26.7, 23.9, 19.4 ppm; IR (neat): 3232, 3101, 3042, 2933, 2864, 2813, 1687, 1640 cm^−1^;

### CO_2_ Absorption in [DBUH][X]

The absorption capacity of CO_2_ was measured according to the standard procedures reported (Wang et al., 2010, 2011). In specific, about 1.0 g [DBUH][X] was added to a 10 ml Schlenk tube which was pre-flushed with CO_2_ gas. With agitation and control of temperature by a thermostat, a stream of CO_2_ was bubbled into [DBUH][X] with a flow rate of 60 ml/min through a stainless steel needle (inner diameter = 10 mm) under atmosphere pressure. The weight of the tube was monitored from time to time until no further increment was detected by an electronic balance with an accuracy of ±0.1 mg. CO_2_ absorption capacity in [DBUH][X] was then calculated based on the mass increasing of the Schlenk tube.

### p*K*_a_ Determinations in [DBUH][OTf]

The UV-vis spectroscopic method was used the p*K*_a_ determination of the substrates involved in this work. The acidity ladder scale and indicator acids, the special UV cell and detailed procedures are similar to the previously reported (Wang et al., 2018a). The concentration of substrate acids was 10^−4^ to 10^−3^ M, the water content of [DBUH][OTf] was less than 100 ppm and the base used in the acidity determination in [DBUH][OTf] was DBU. The p*K*_a_ for each substrate was the average of 3 individual experiments, and the standard deviation (SD) is less than ±0.05 p*K* units.

## Conclusions

In summary, we synthesized 11 DBU-based PILs with different basicity in [DBUH][OTf] and systematically investigated their CO_2_ absorption kinetic and capacity in these PILs. The basicity scale for the anion of these PILs in [DBUH][OTf] was established by extrapolation or direct determination. The CO_2_ absorption in the weakly basic PILs are slow and practically have negligible absorption capacity, which is in line with a physical absorption mechanism. On the other hand, faster rates and higher absorption capacity were observed in the strongly basic PILs, and the spectroscopic studies support a chemical absorption mechanism in these PILs. The correlation between CO_2_ absorption capacities and basicities of PILs, excluding those very weakly basic ones, in [DBUH][OTf] shows an excellent linear relationship, which indicates that the basicity of anion dictates the absorption ability and mechanism. We hope these results can be of help for a better understanding of structural implication of PILs on the CO_2_ absorption, and also for a rational design of PILs in this connection.

## Author Contributions

PJ and J-PC conceived and designed the experiments and supervised the project; FG and ZW performed the experiments; FG, PJ, and J-PC prepared and revised the manuscript.

### Conflict of Interest Statement

The authors declare that the research was conducted in the absence of any commercial or financial relationships that could be construed as a potential conflict of interest.

## Footnotes

^1^From the correlation equation for N^+^-H series (Figure 2), the autoprotolysis constant of [DBUH][OTf], ${\text{p}K}_{\text{auto}}^{\left[\text{DBUH}\right]\left[\text{OTf}\right]}$ = [DBU]•[HOTf] = 14.5 can also be derived as follow: *K*_a_ = [DBU][H^+^]/[DBUH^+^] = $K_{\text{auto}}^{\left[\text{DBUH}\right]\left[\text{OTf}\right]}$/[DBUH^+^], therefore: $K_{\text{auto}}^{\left[\text{DBUH}\right]\left[\text{OTf}\right]}$ = [DBUH^+^]•*K*_a_, the molar concentration of [DBUH][OTf] at 25 °C (4.45 mol/L) can be calculated from the density [1.345 g/cm^3^ at 25°C (Miran et al., 2012)] and molecular weight (302.3 g/mol) of [DBUH][OTf]. p$K_{\text{auto}}^{\left[\text{DBUH}\right]\left[\text{OTf}\right]}$ is about 0.5 or 4.5 p*K* units higher than that of water or EAN [p$K_{\text{auto}}^{\text{EAN}}$ = 10.0 (Kanzaki et al., 2016)], respectively.

^2^Although in some cases, there do exist discrepancies between nucleophilicity and basicity of nucleophile and the traditional Brönsted analysis fails (Mayr and Ofial, 2016). However, recent studies show that linear correlations were found between basicity and nucleophilicity of N-heterocyclic carbenes (NHCs) in the reactions that involving CO_2_ as the substrate (Niu et al., 2017; Wang et al., 2018,b).
