# Supplementary material for: CO2 Absorption by DBU-Based Protic Ionic Liquids: Basicity of Anion Dictates the Absorption Capacity and Mechanism
Source: Front Chem. 2019 Jan 17;6:658. doi: 10.3389/fchem.2018.00658 (PMC6344442; doi:10.3389/fchem.2018.00658)
Supplement: Supplementary file 1 [file Data_Sheet_1.docx]

Supplementary Material

CO_2_ Absorption by DBU-Based Protic Ionic Liquids: Basicity of Anion Dictates the Absorption Capacity and Mechanism

Feixiang Gao,^1,+^ Zhen Wang,^3,+^ Pengju Ji^1,*^ and Jin-Pei Cheng^1,2,*^

^1^Center of Basic Molecular Science (CBMS), Department of Chemistry, Tsinghua University, Beijing, 100084, China

^2^State Key Laboratory of Elemento-Organic Chemistry, Collaborative Innovation Centre of Chemical Science and Engineering, Nankai University, Tianjin, 300071, China

^3^School of Chemical and Environmental Engineering, Anyang Institute of Technology, Anyang, 455000, China

^+^These authors contribute equally to this work;
[jipengju@mail.tsinghua.edu.cn;](mailto:jipengju@mail.tsinghua.edu.cn(Ji.P.))  [jinpei_cheng@mail.tsinghua.edu.cn;](mailto:jinpei_cheng@mail.tsinghua.edu.cn(Cheng)

# Synthesis of DBU-based PILs [DBUH][X].

Figure S1. The synthesis of DBU-based PILs (**1**-**8**) through method A

Method A: These PILs (**1**-**8**, Figures 1 and S1) were synthesized by a direct equimolar acid and base neutralization reaction under neat condition. [Wang et al., 2010] In specific, 0.1 mol DBU (1,5-diazabicyclo[5.4.0]-5-undecene) and 0.1 mol acid HX were added slowly to a 250 ml round-bottom flask in a glove-box, then the reaction mixture was stirred at 60 ^o^C for 24 hours under argon protection. The crude PILs were dried in vacuo at a constant temperature of 60 ^o^C for overnight, then flushed with argon and stored in a glove-box before used as the media for CO_2_ absorption.

Figure S2. The synthesis of DBU-based PILs (**9**-**11**) through method B

Method B: These PILs (**9**-**11**, Figures 1 and S2) were synthesized by direct equimolar acid and base neutralization reactions in MeOH, [Losetty et al., 2017] instead of under neat condition as described in Method A. After reaction, the methanol was removed under vacuum. The crude PILs were dried in vacuo at a constant temperature of 40 ^o^C for overnight, then flushed with argon and stored in a glove-box before used as the media for CO_2_ absorption.

# NMR and IR spectra

- [DBUH][Im] **(1)**

**
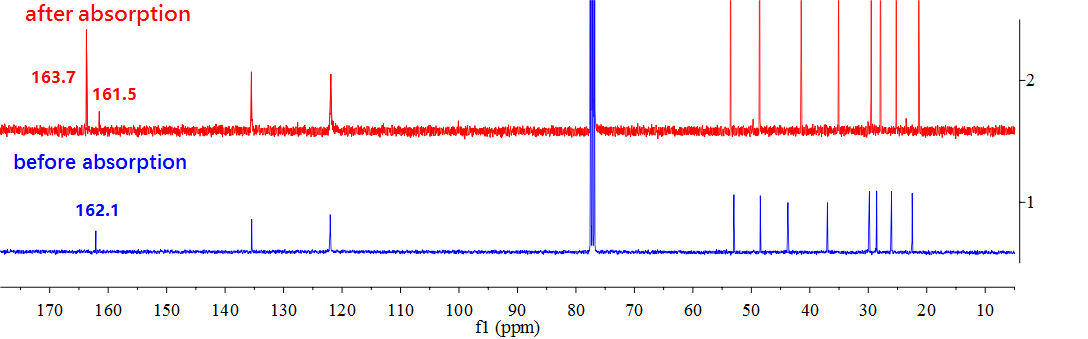
**

**
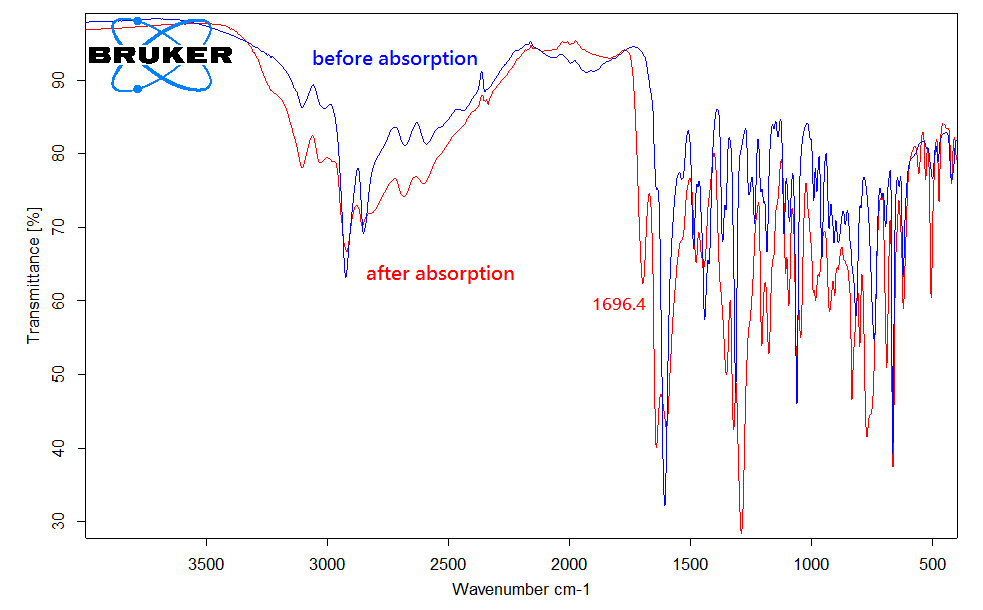
**

Figure S3. ^13^C NMR and IR spectra for [DBUH][Im] (**1**) before and after CO_2_ absorption


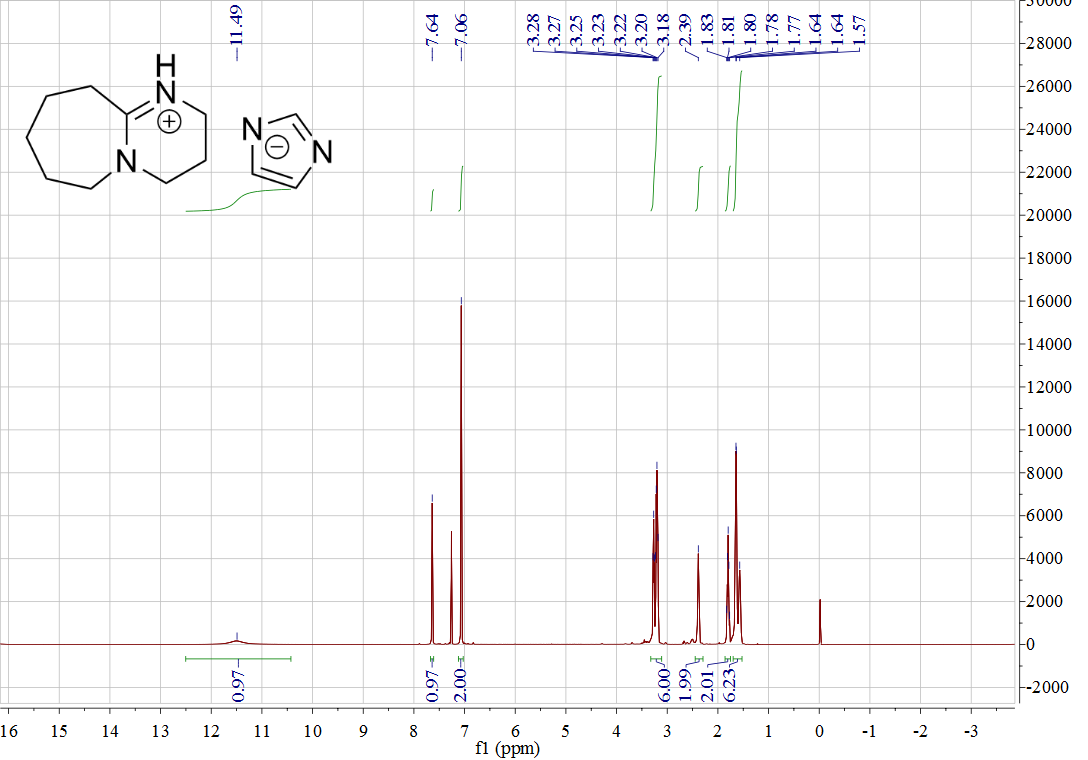


Figure S4. ^1^H NMR of [DBUH][Im] (**1**)


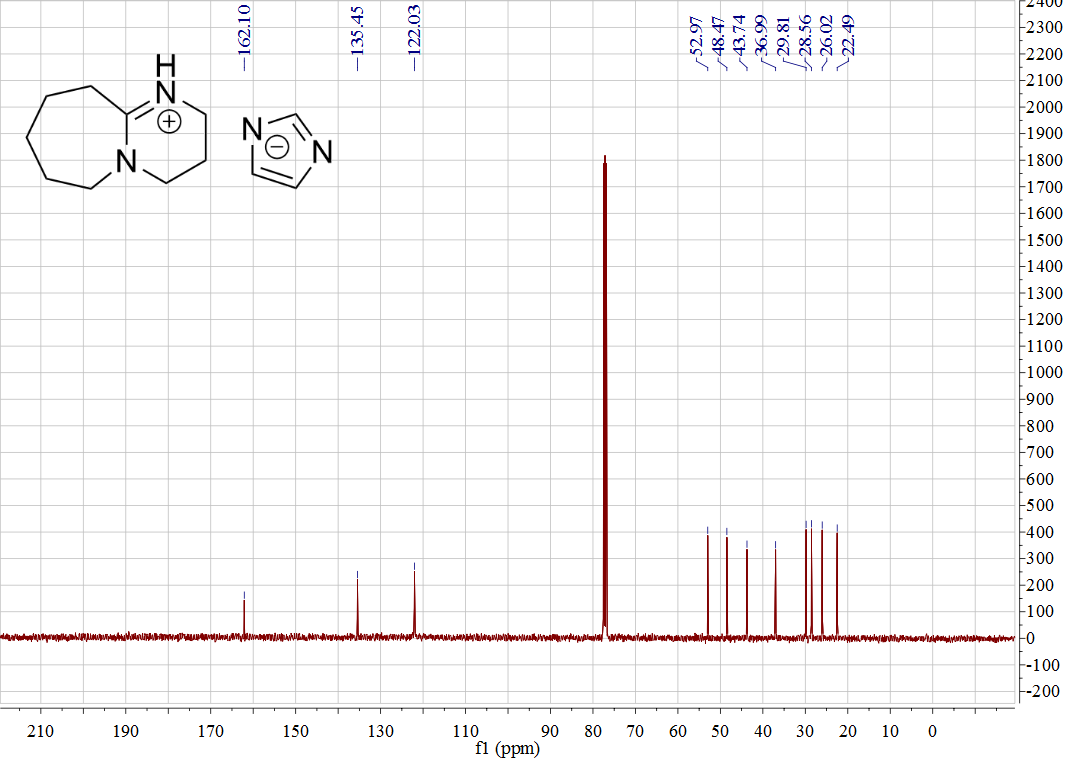


Figure S5: ^13^C NMR of [DBUH][Im] (**1**)

- [DBUH][Pyra] (**2**)


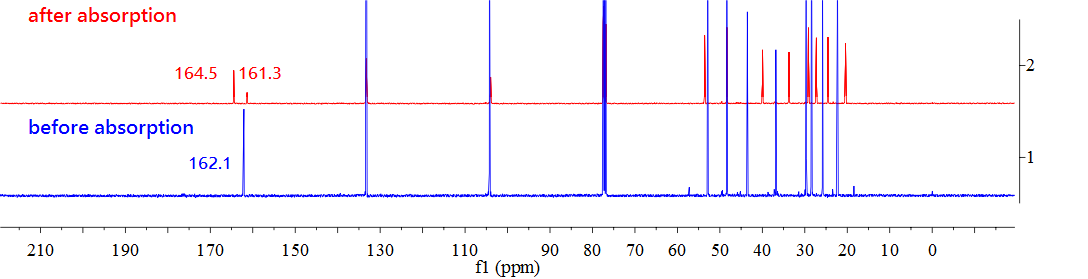


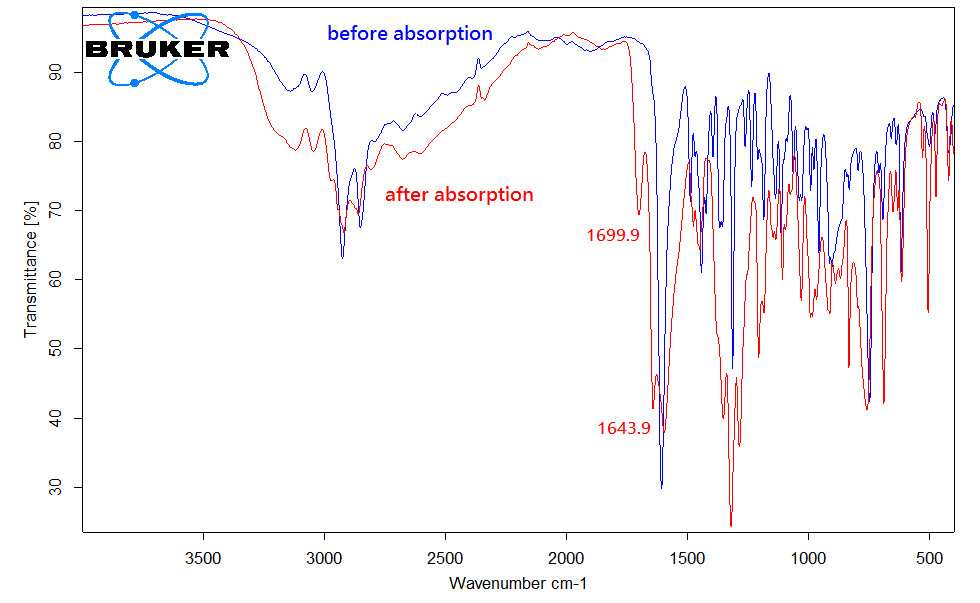


Figure S6. ^13^C NMR and IR spectra for [DBUH][Pyra] (**2**) before and after CO_2_ absorption


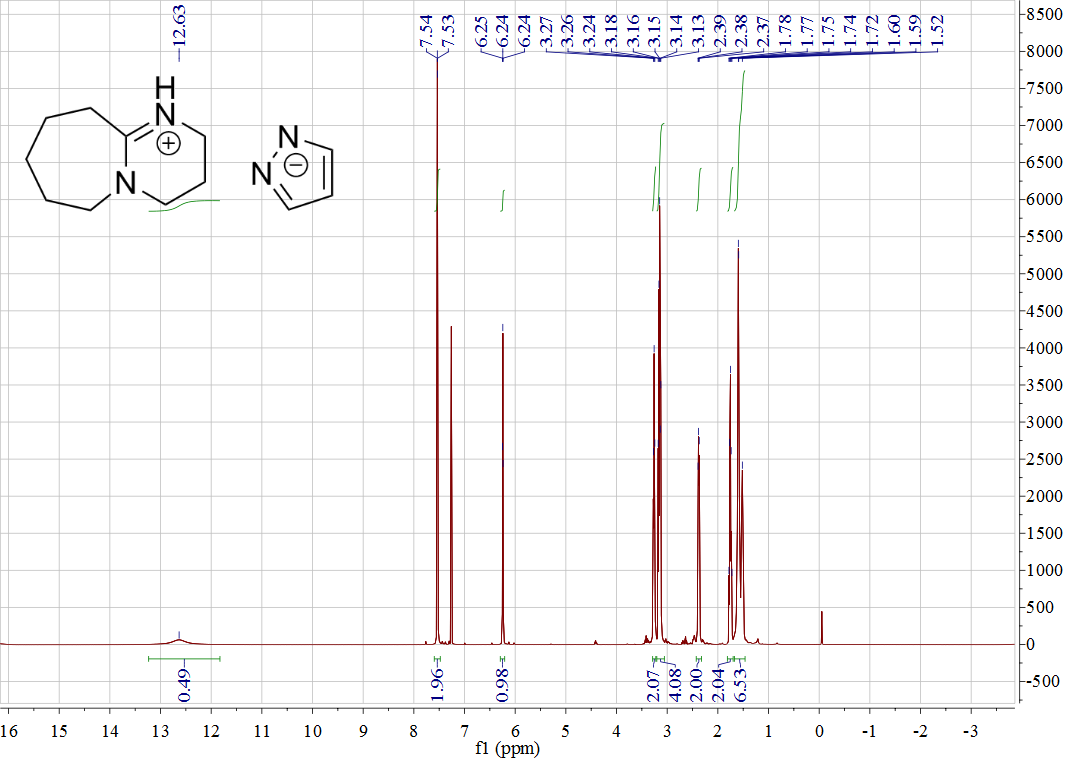


Figure S7. ^1^H NMR of [DBUH][Pyra] (**2**)


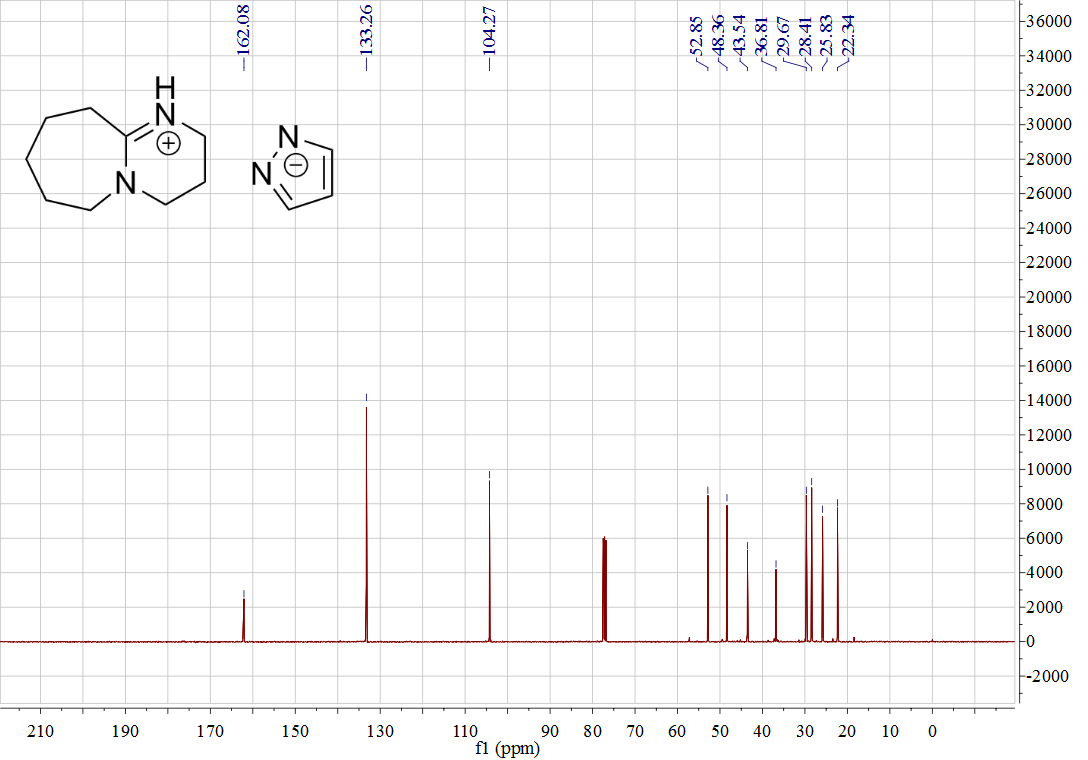


Figure S8. ^13^C NMR of [DBUH][Pyra] (**2**)

- [DBUH][4-MP] (**3**)


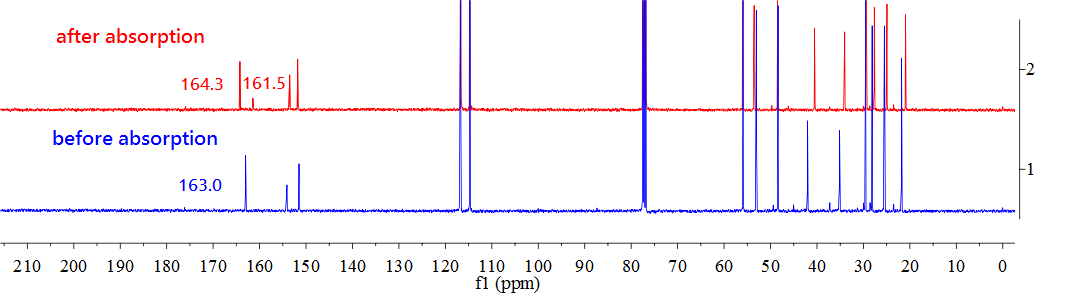


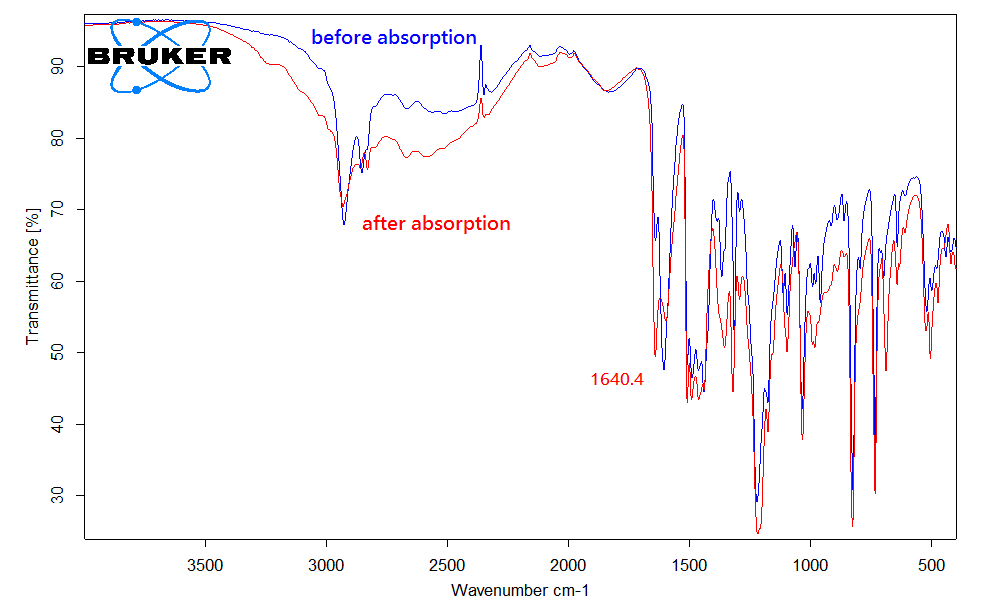


Figure S9. ^13^C NMR and IR spectra for [DBUH][4-MP] (**3**) before and after CO_2_ absorption


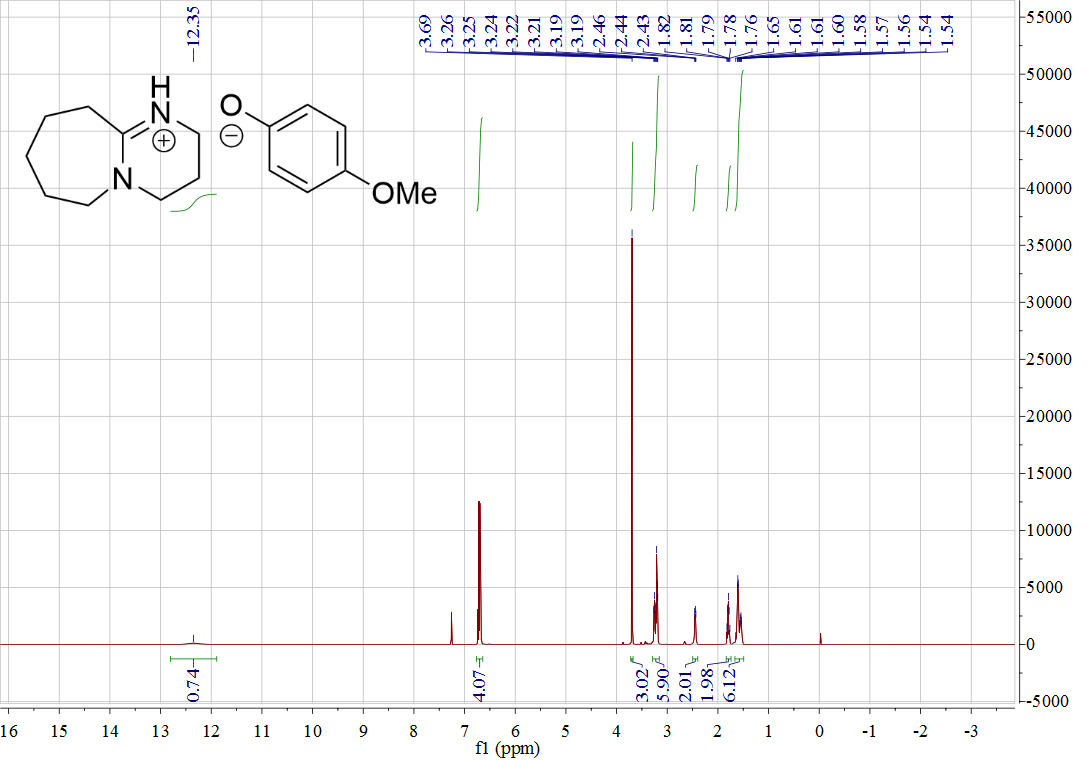


Figure S10. ^1^H NMR of [DBUH][4-MP] (**3**)


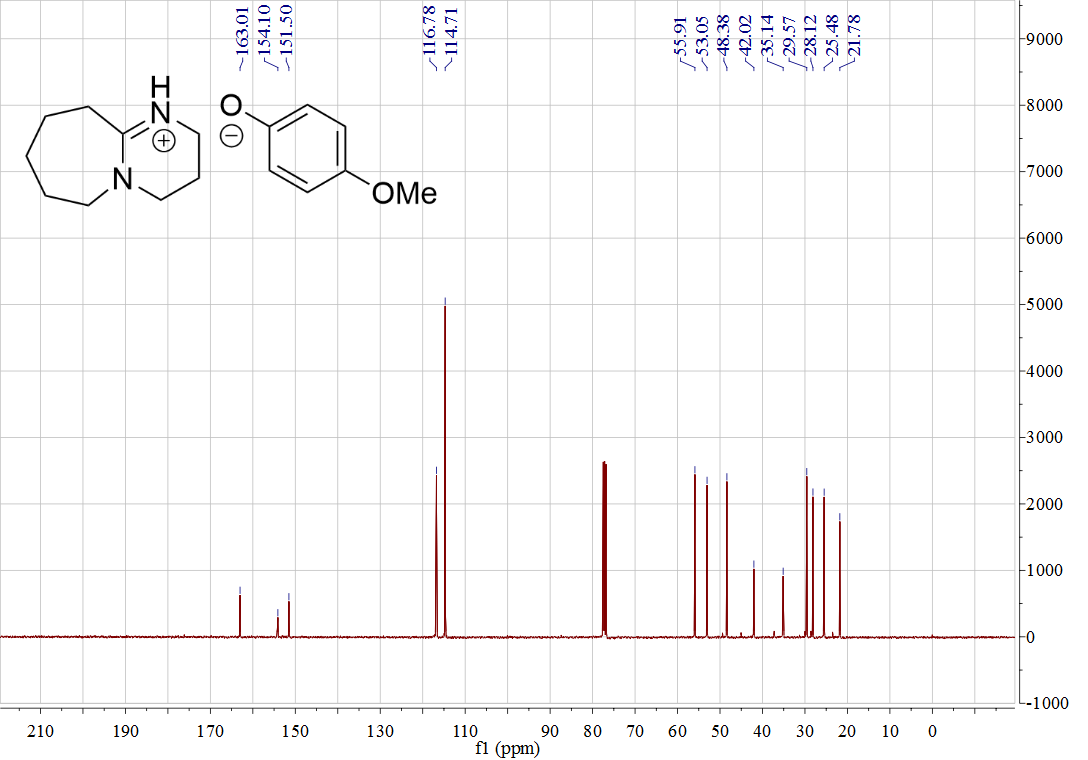


Figure S11. ^13^C NMR of [DBUH][4-MP] (**3**)

- [DBUH][PhO] (**4**)


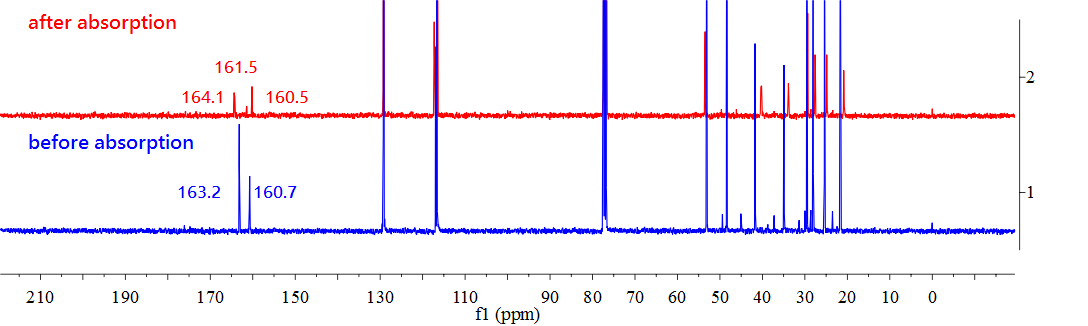


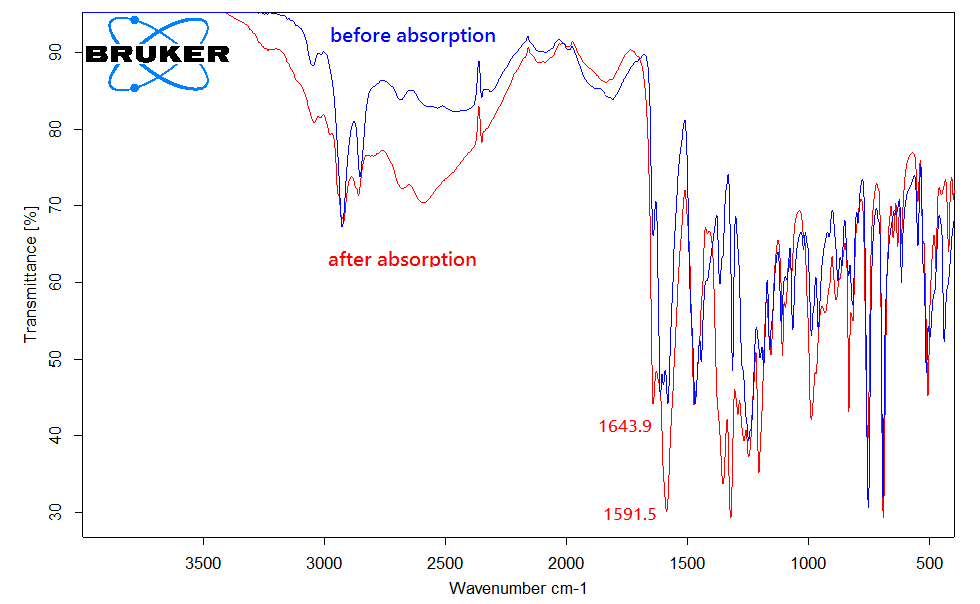


Figure S12. ^13^C NMR and IR spectra for [DBUH][PhO] (**4**) before and after CO_2_ absorption


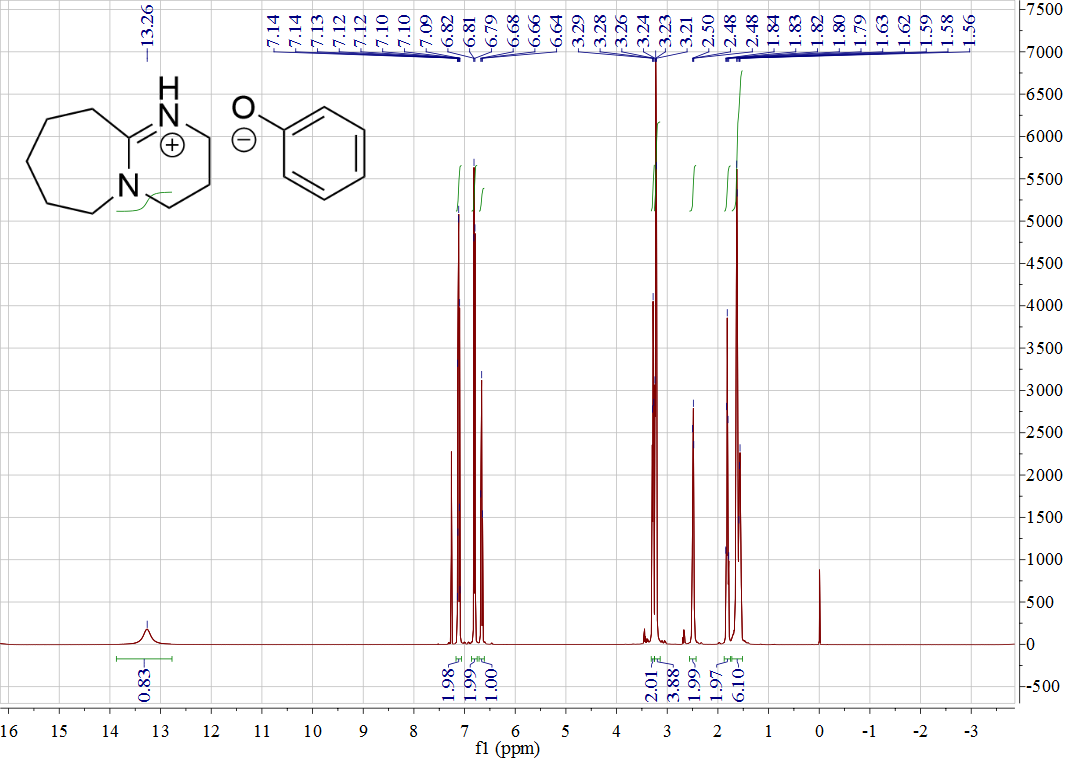


Figure S13. ^1^H NMR of [DBUH][PhO] (**4**)


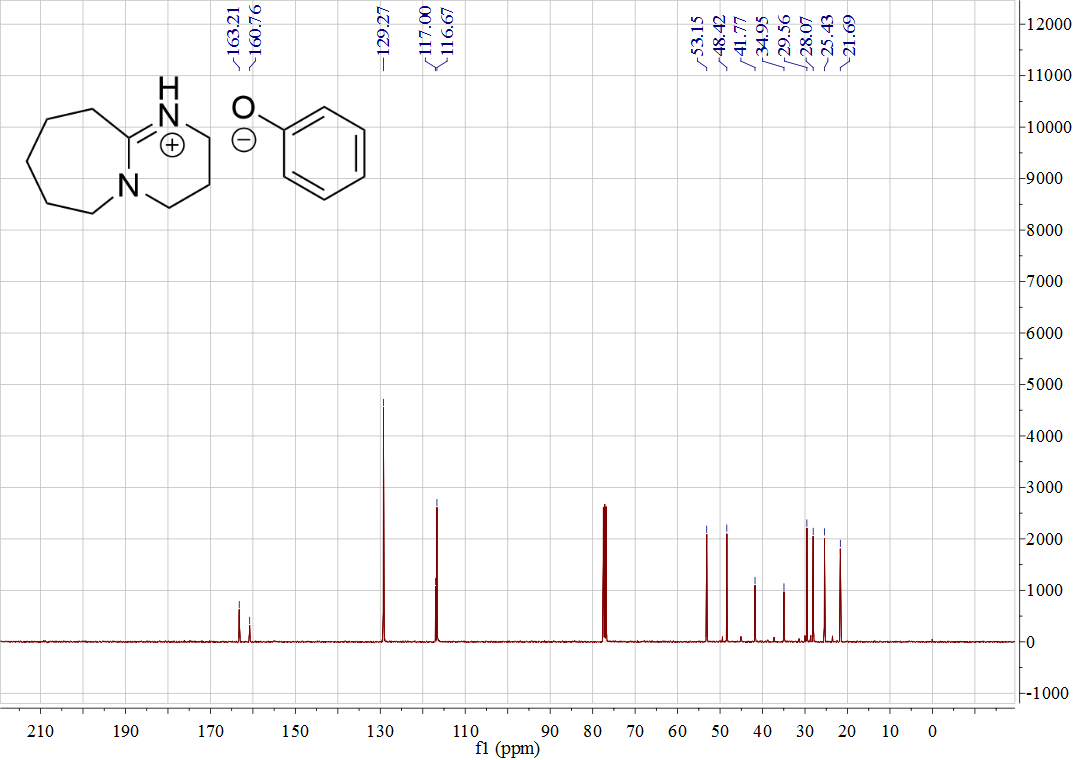


Figure S14. ^13^C NMR of [DBUH][PhO] (**4**)

- [DBUH][4-BP] (**5**)


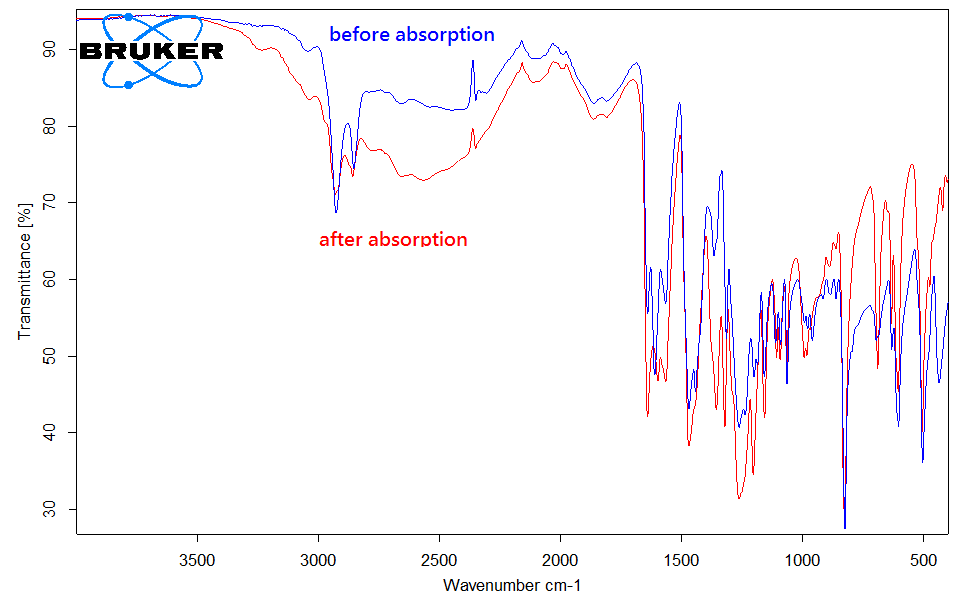


Figure S15. IR spectrum for [DBUH][BP] (**5**) before and after CO_2_ absorption


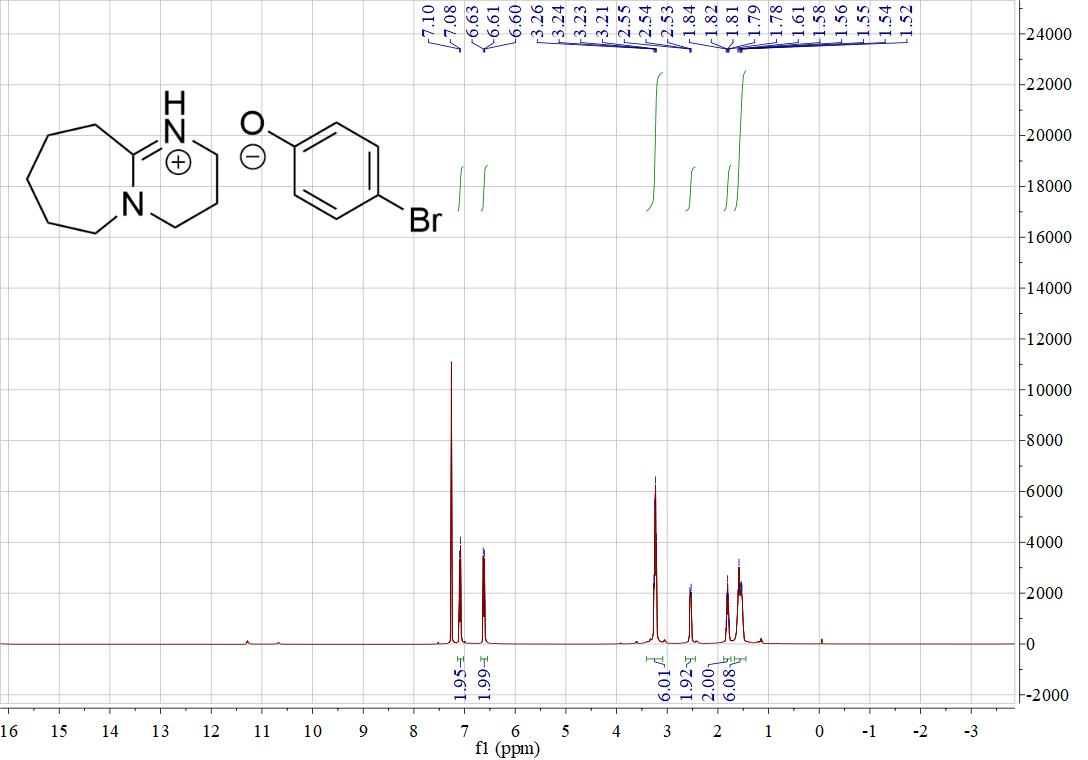


Figure S16. ^1^H NMR of [DBUH][4-BP] (**5**)


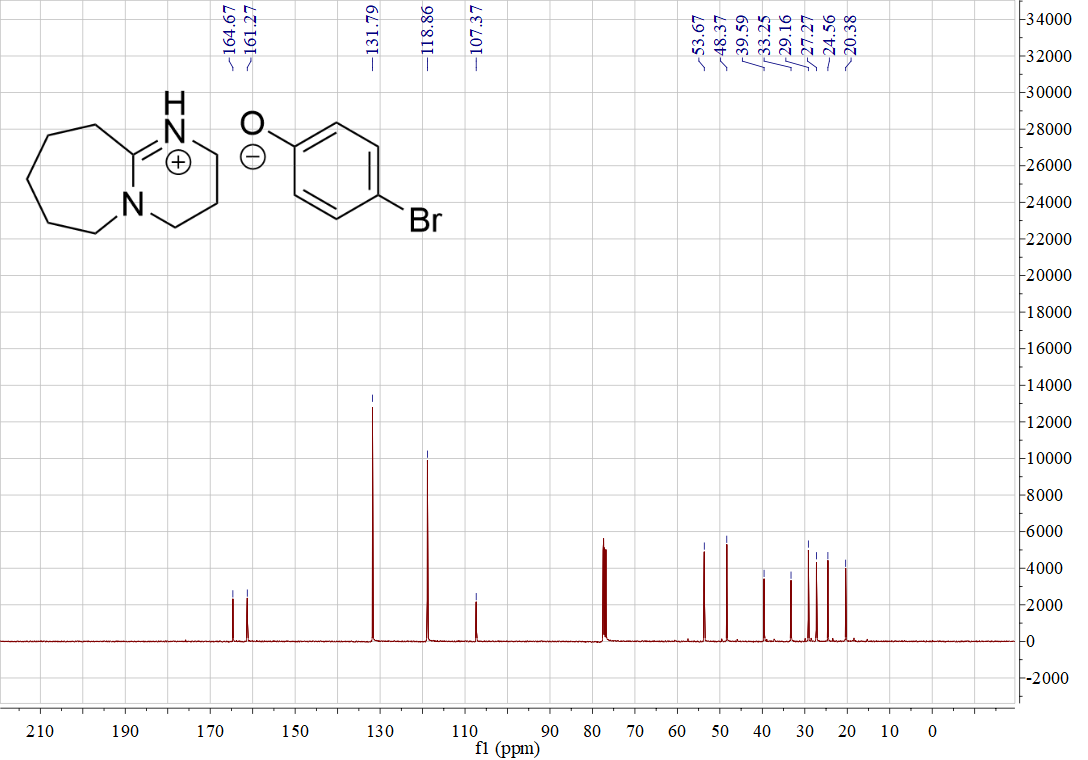


Figure S17. ^13^C NMR of [DBUH][4-BP] (**5**)

- [DBUH][4-TFMP] (**6**)


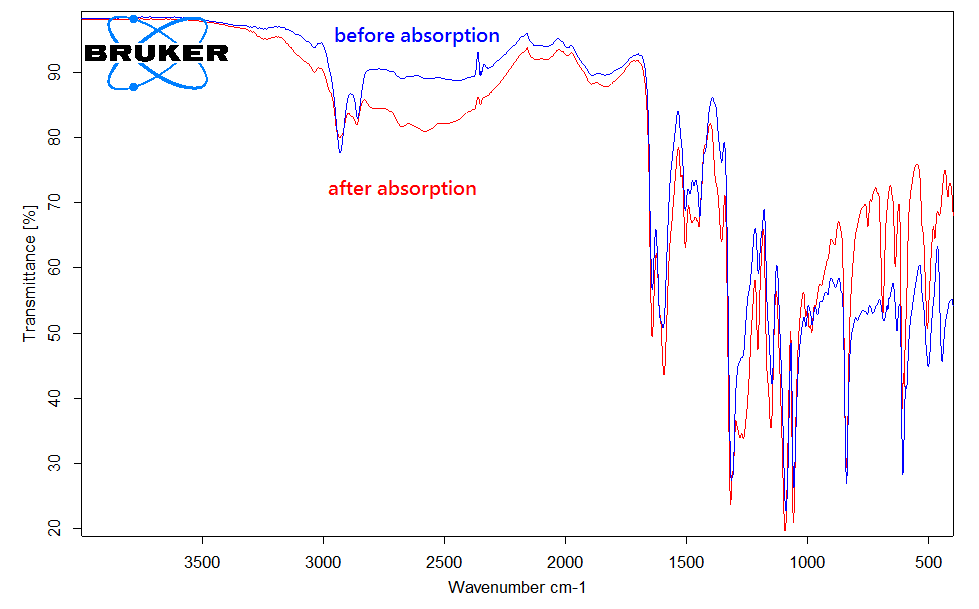


Figure S18. IR spectrum for [DBUH][TFMP] (**6**) before and after CO_2_ absorption


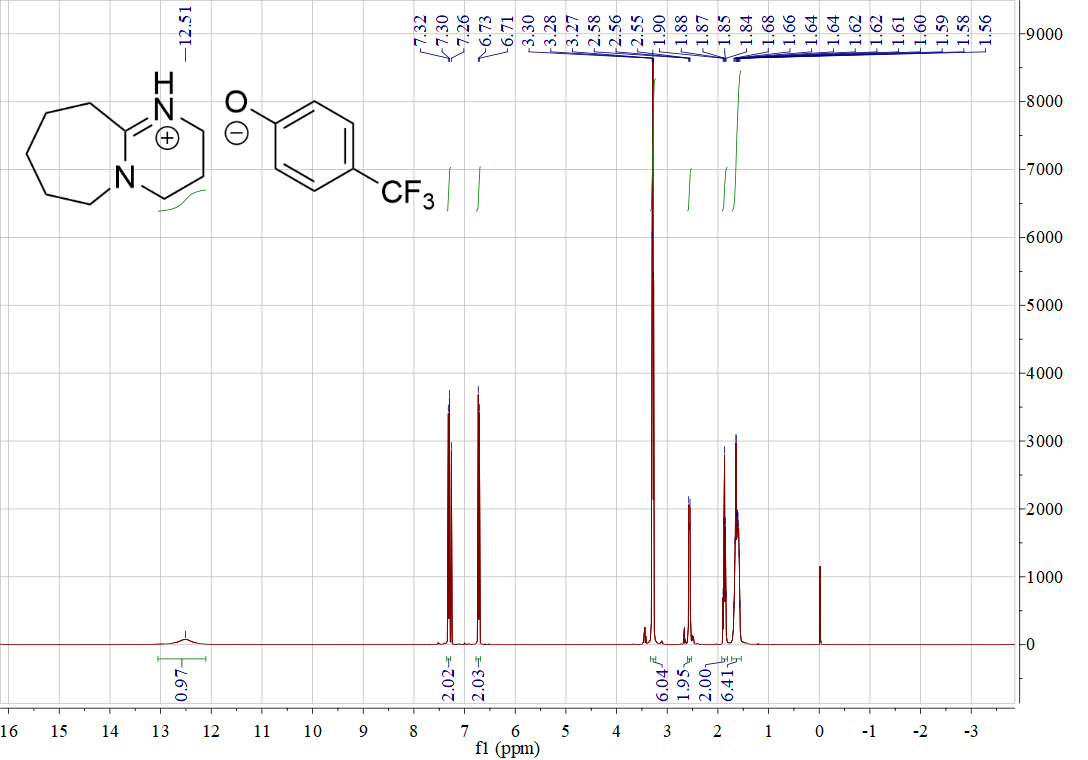


Figure S19. ^1^H NMR of [DBUH][4-TFMP] (**6**)


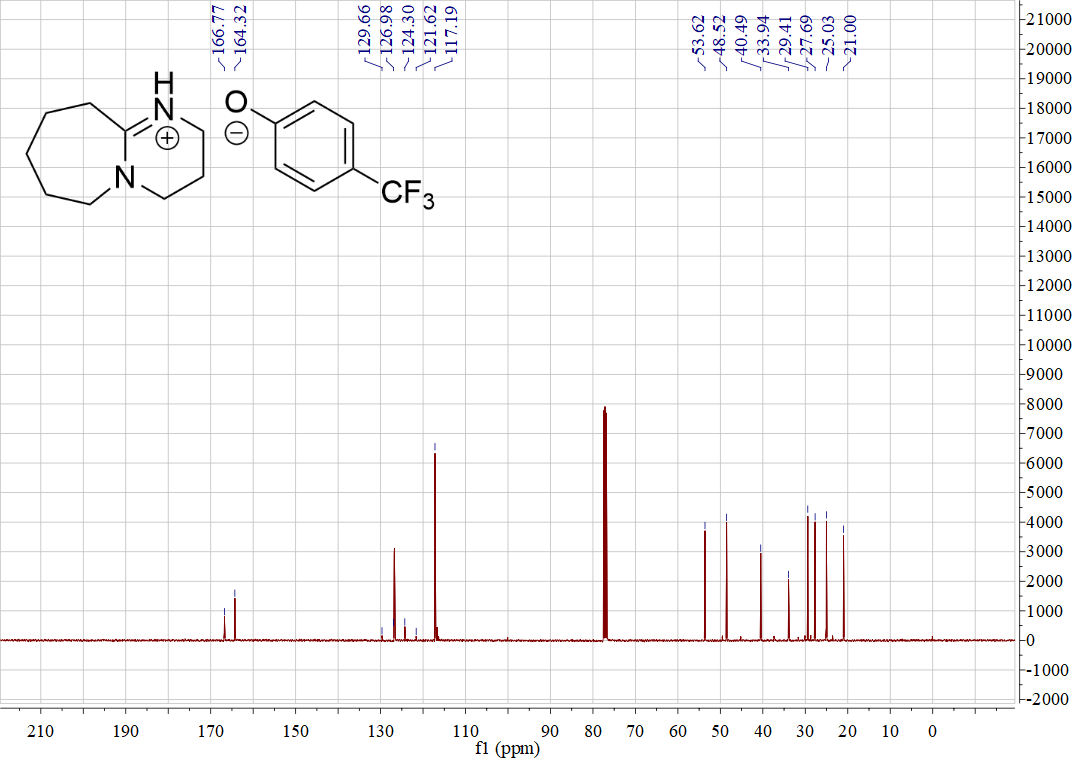


Figure S20. ^13^C NMR of [DBUH][4-TFMP] (**6**)

- [DBUH][1,2,4-Triz] (**7**)


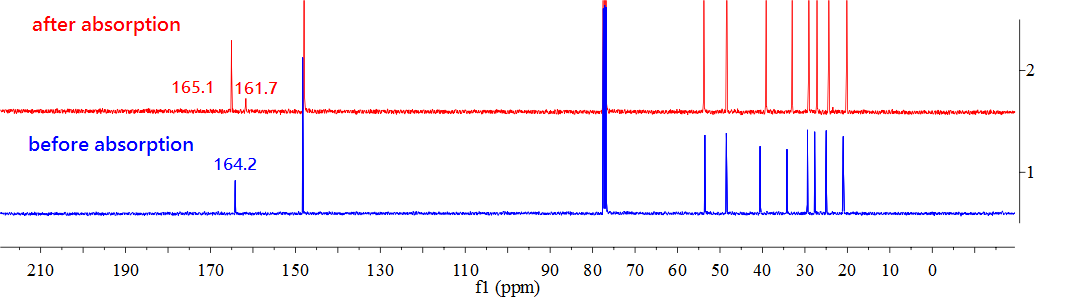


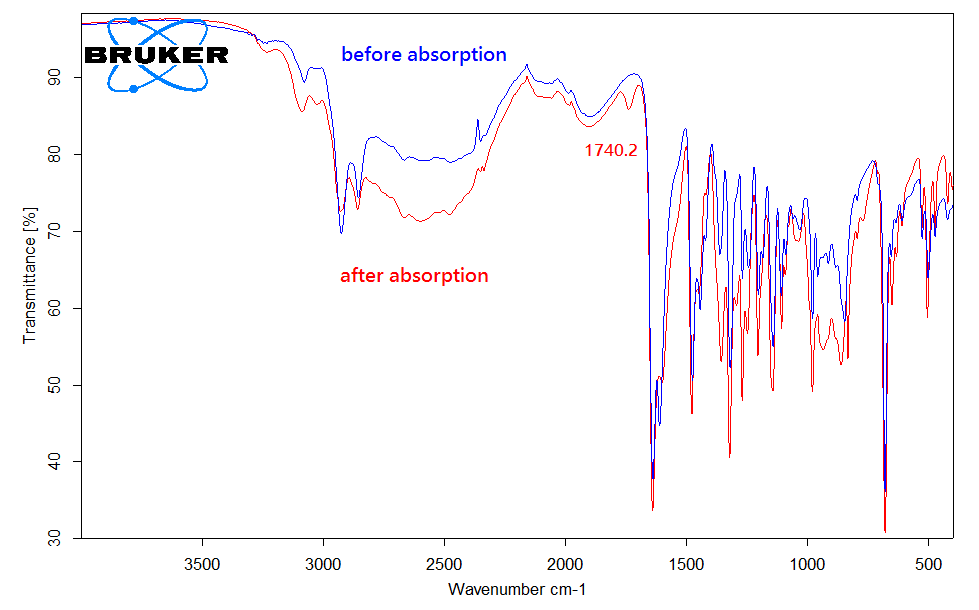


Figure S21. ^13^C NMR and IR spectra for [DBUH][1,2,4-Triz] (**4**) before and after CO_2_ absorption


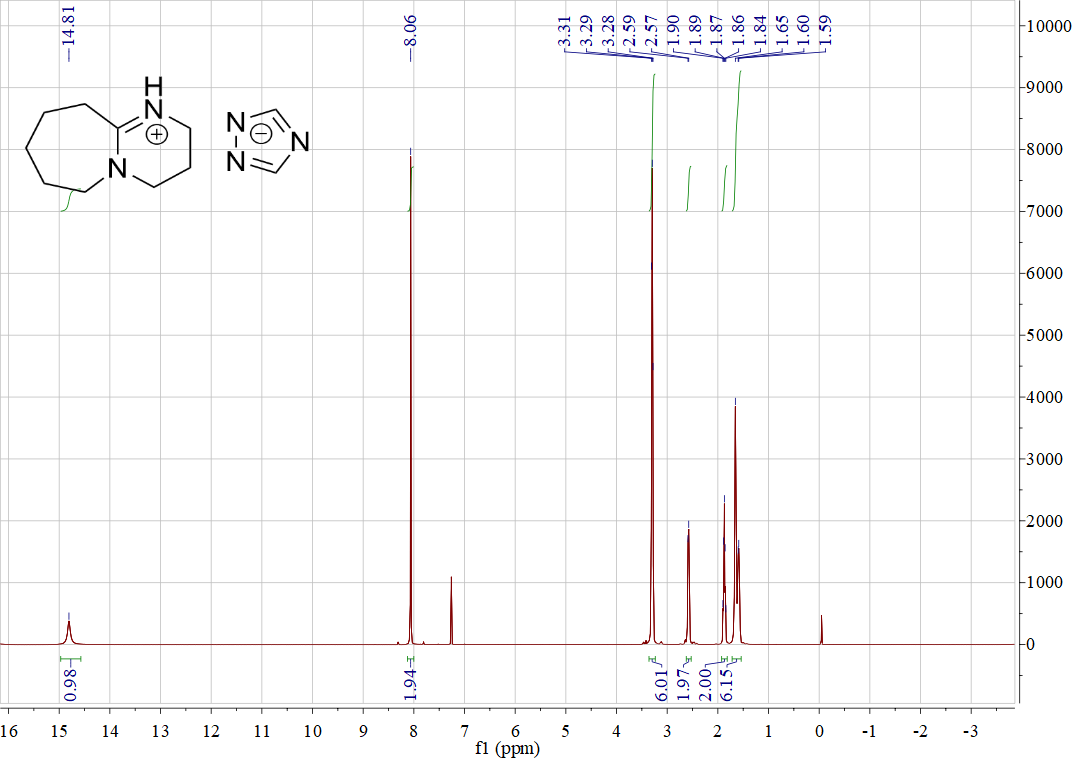


Figure S22. ^1^H NMR of [DBUH][1,2,4-Triz] (**7**)


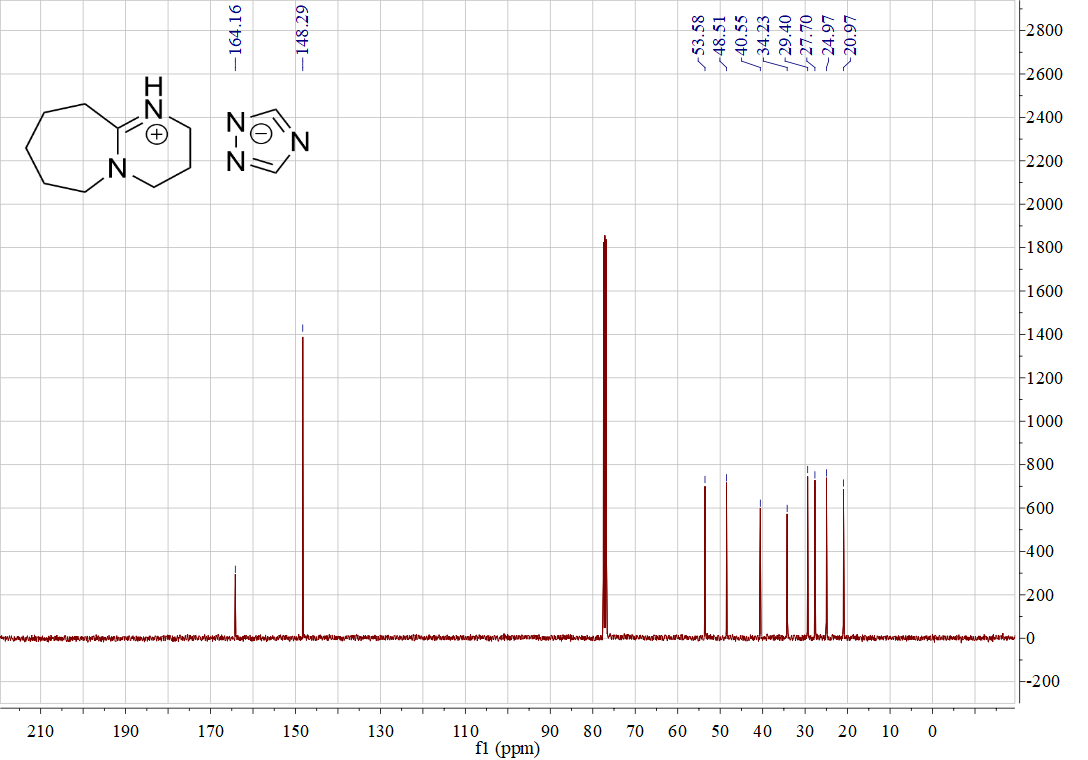


Figure S23. ^13^C NMR of [DBUH][1,2,4-Triz] (**7**)

- [DBUH][1,2,3-Triz] (**8**)


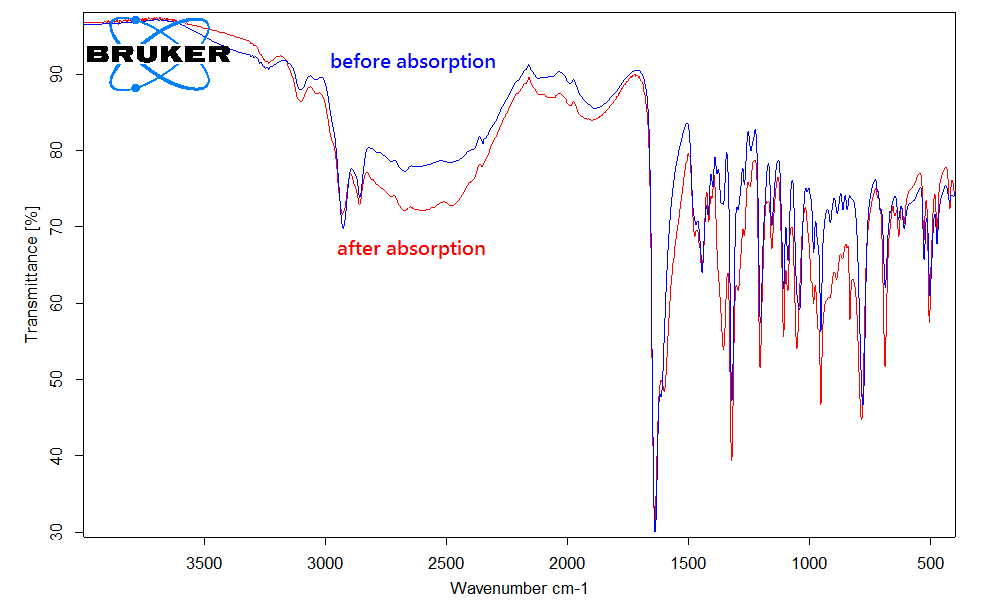


Figure S24. IR spectra for [DBUH][1,2,3-Triz] (**8**) before and after CO_2_ absorption


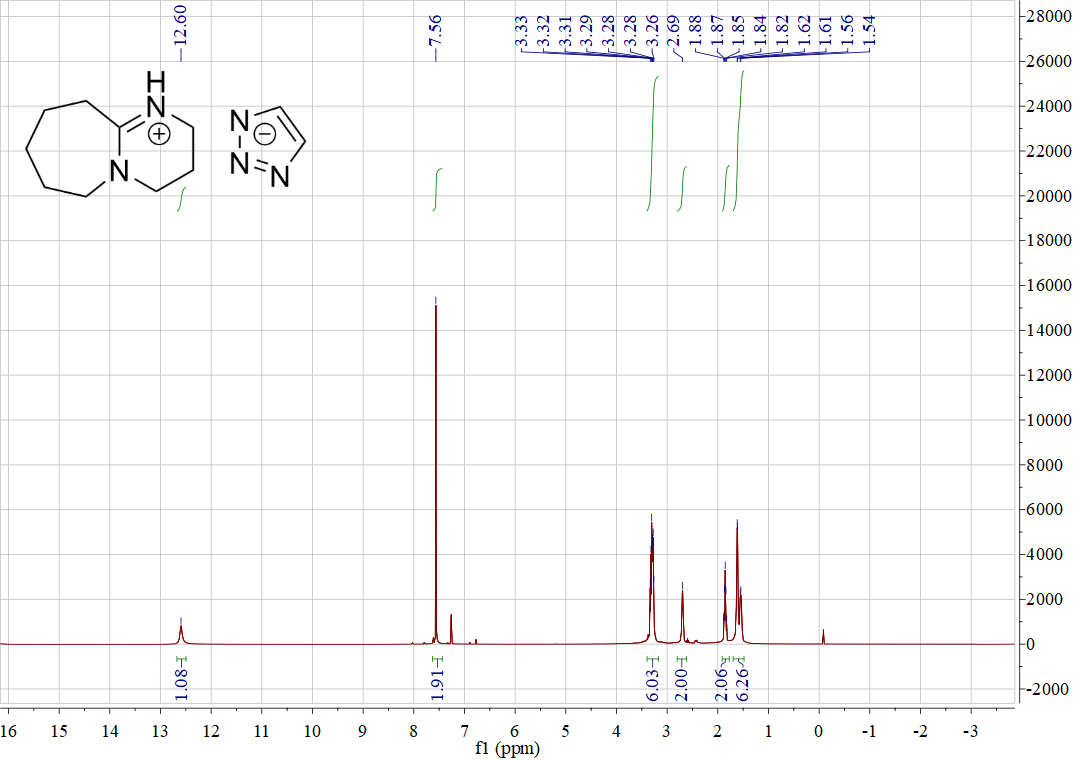


Figure S25. ^1^H NMR of [DBUH][1,2,3-Triz] (**8**)


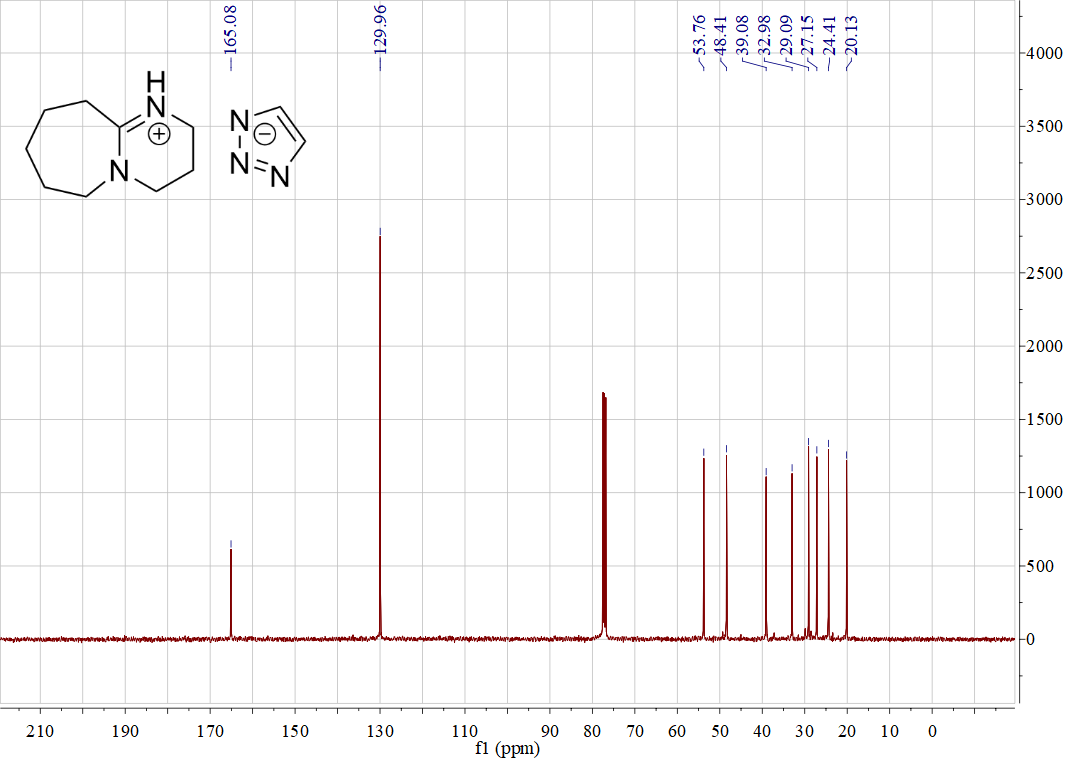


Figure S26. ^13^C NMR of [DBUH][1,2,3-Triz] (**8**)

- [DBUH][OAc] (**9**)


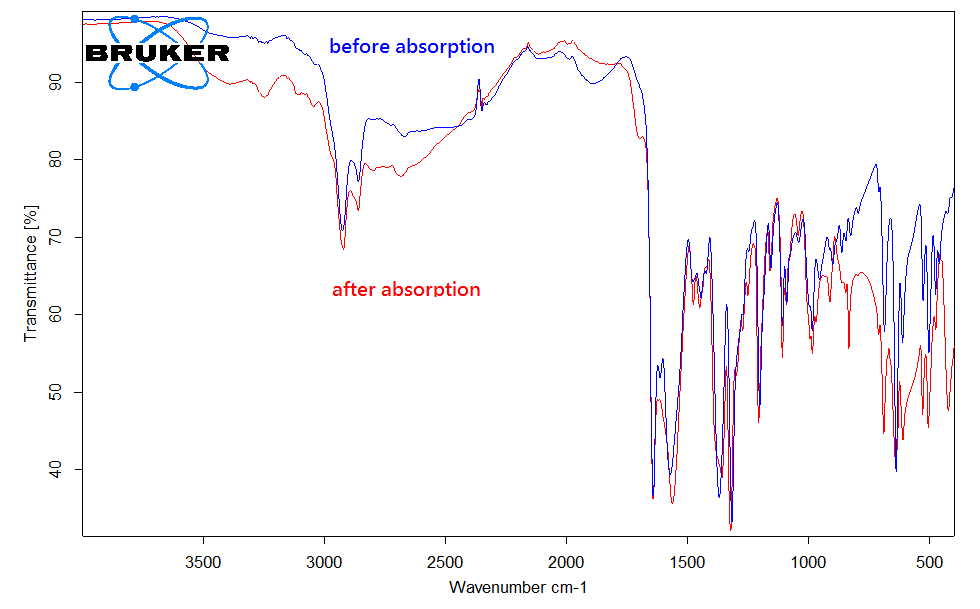


Figure S27. IR spectra for [DBUH][OAc] (**9**) before and after CO_2_ absorption


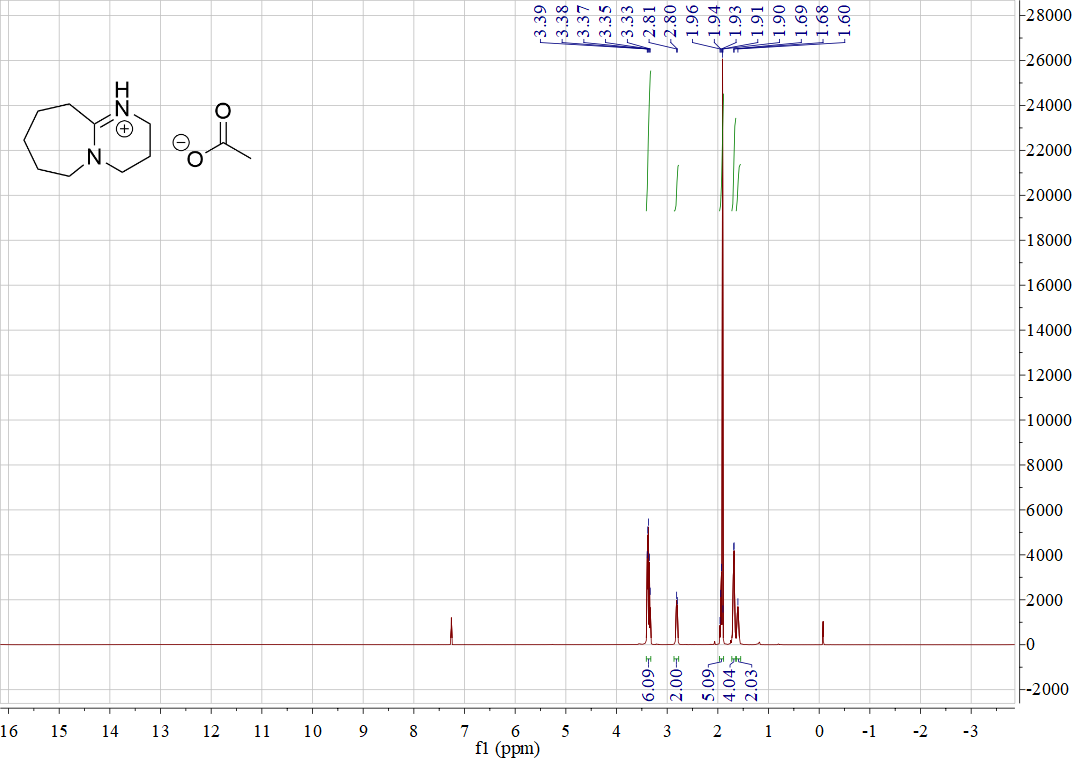


Figure S28. ^1^H NMR of [DBUH][OAc] (**9**)


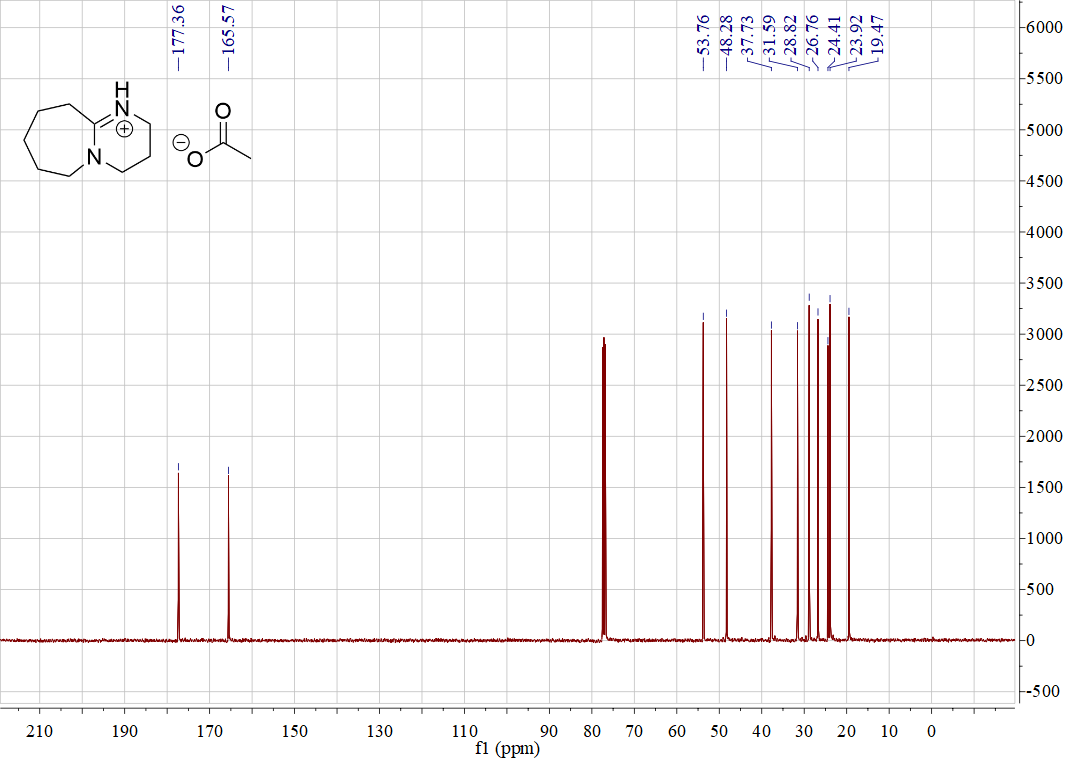


Figure S29. ^13^C NMR of [DBUH][OAc] (**9**)

- [DBUH][Cl_2_CHCOO] (**10**)


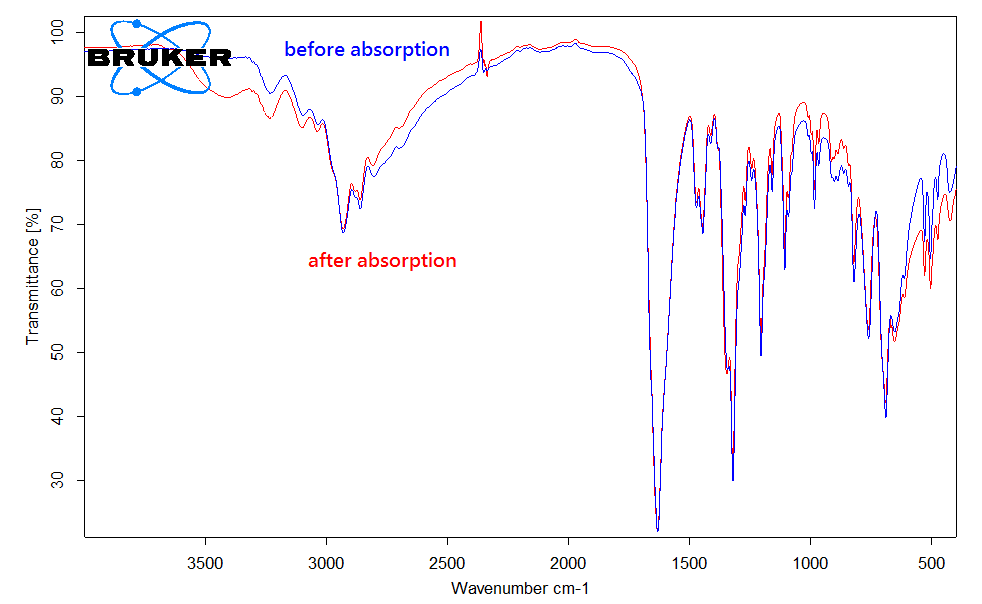


Figure S30. IR spectra for [DBUH][Cl_2_CHCOO] (**10**) before and after CO_2_ absorption

**
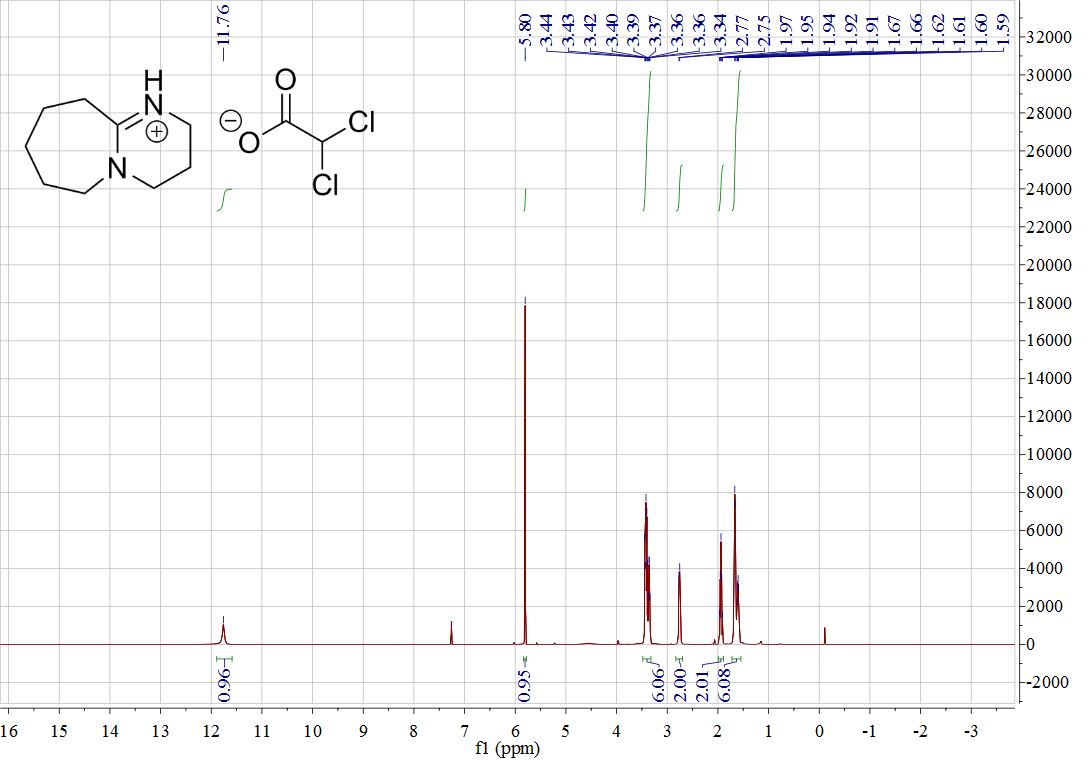
**

Figure S31. ^1^H NMR of [DBUH][Cl_2_CHCOO] (**10**)

**
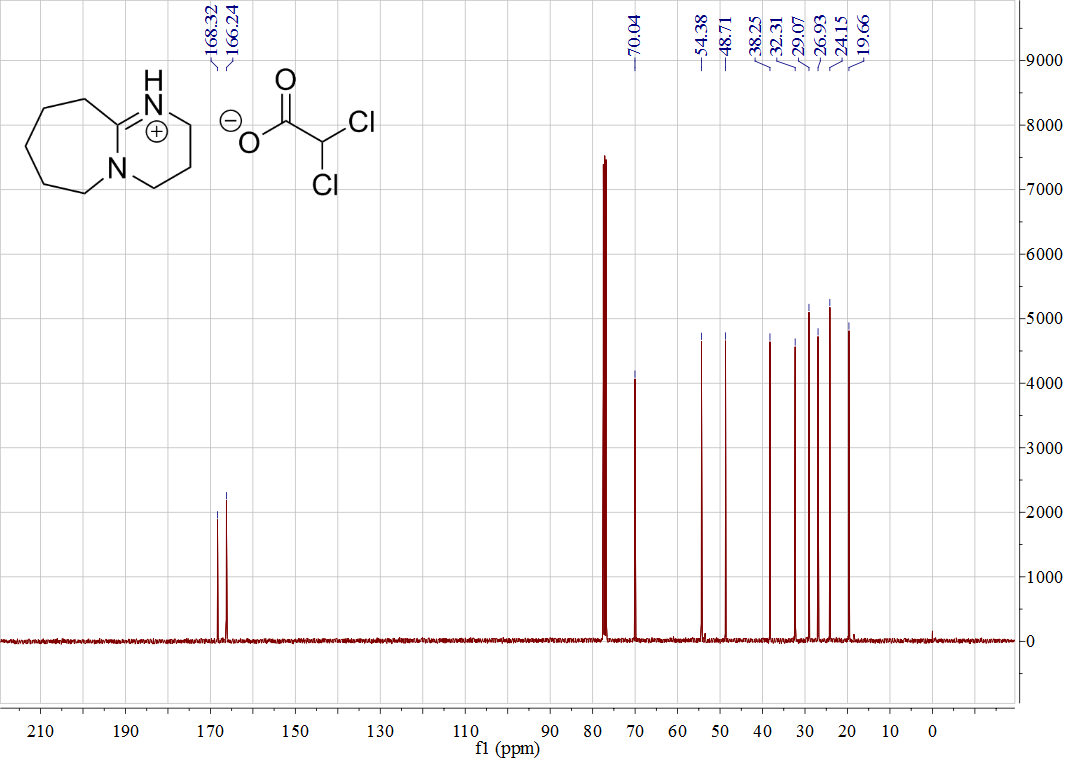
**

Figure S32. ^13^C NMR of [DBUH][Cl_2_CHCOO] (**10**)

- [DBUH][TFA] (**11**)

**
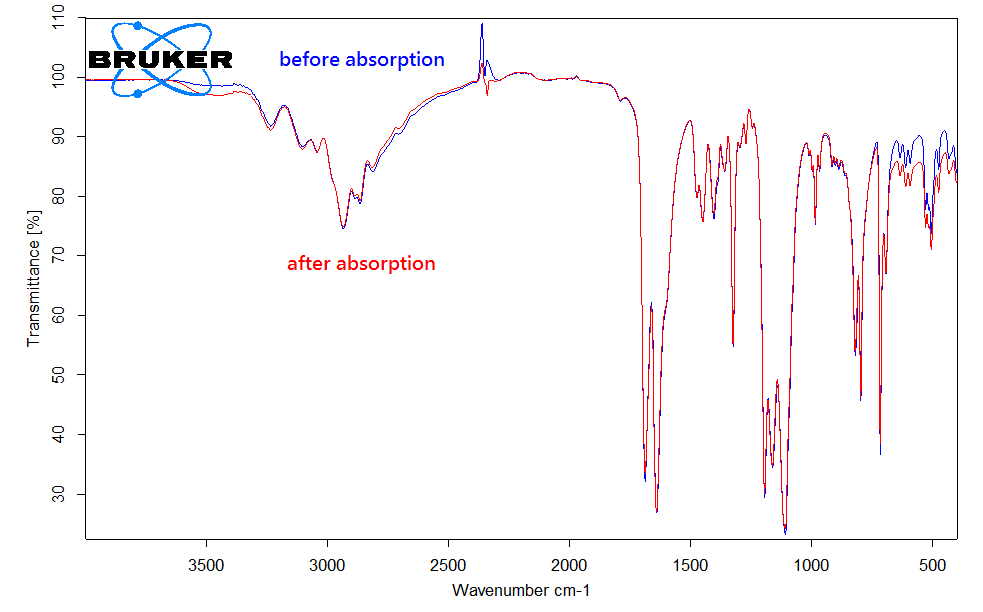
**

Figure S33. IR spectra for [DBUH][TFA] (**11**) before and after CO_2_ absorption

**
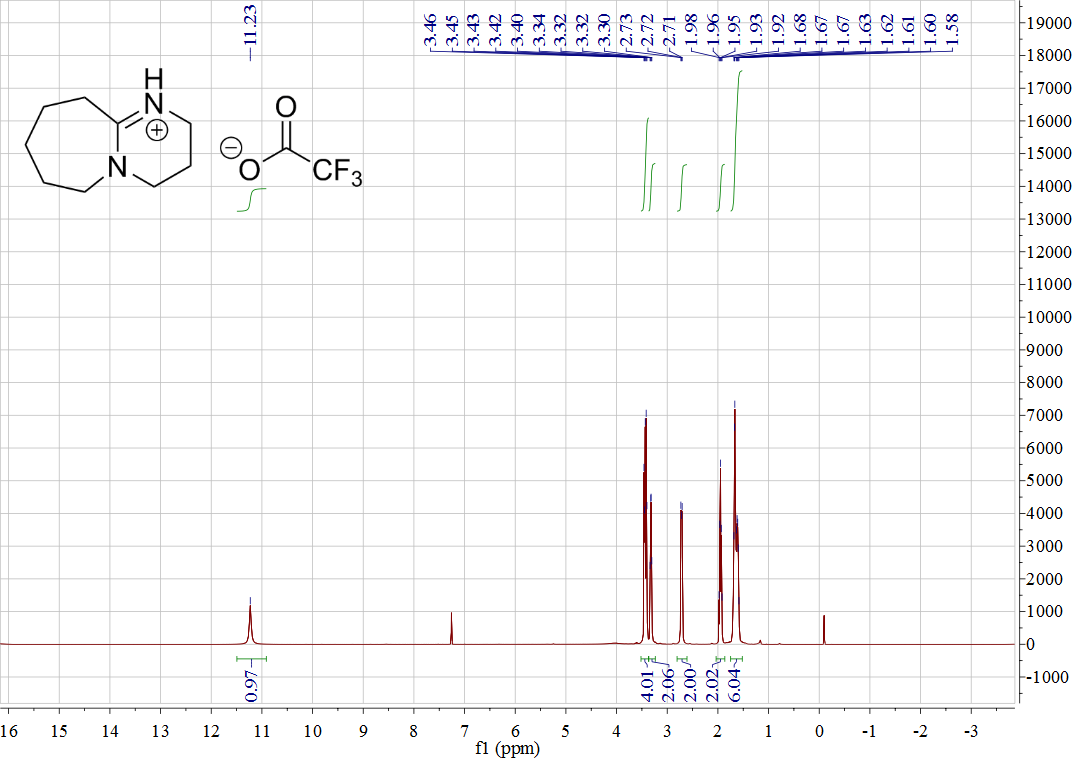
**

Figure S34. ^1^H NMR of [DBUH][TFA] (**11**)

**
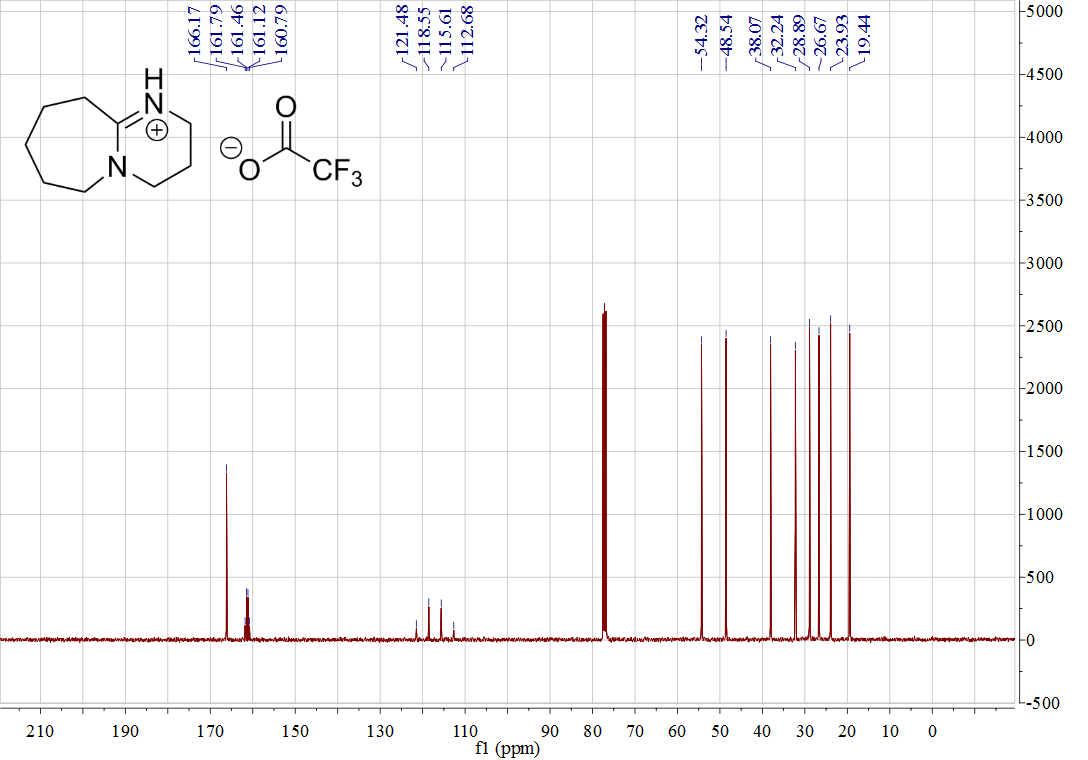
**

Figure S35. ^13^C NMR of [DBUH][TFA] (**11**)

# Linear correlations between anion basicity and absorption capacity.

Figure S36. Correlation between the anion basicity in water^[3]^ and absorption capacity in [DBUH][X] (**1**-**9**)

Figure S37. Correlation between the anion basicity in DMSO^[3]^ and absorption capacity in [DBUH][X] (**1**-**9**)

# References.

[1] Wang, C. M., Luo, H. M., Jiang, D. E., Li, H. R., Dai, S. (2010). Carbon dioxide capture by superbase-derived protic ionic liquids. *Angew. Chem. Int. Ed.* 49, 5978-5981. doi: 10.1002/anie.201002641

[2] Losetty, V., Matheswaran, P., Wilfred, C. D. (2017). Synthesis, thermophysical properties and COSMO-RS study of DBU based protic ionic liquids. *J. Chem. Thermodyn.* 105, 151-158. doi:10.1016/j.jct.2016.10.021

[3] p*K*_a_ data in molecular solvents is available at *i*Bond 2.0 Database: ibond.chem.tsinghua.edu.cn; ibond.nankai.edu.cn.
